# Bispecific Antibodies in Solid Tumors: Advances and Challenges

**DOI:** 10.3390/ijms26125838

**Published:** 2025-06-18

**Authors:** Khine Swe Shan, Saba Musleh Ud Din, Shivani Dalal, Teresita Gonzalez, Misha Dalal, Pablo Ferraro, Atif Hussein, Michel Vulfovich

**Affiliations:** 1 Memorial Health Care, Division of Hematology and Oncology, Pembroke Pines, FL 33028, USA; sdalal@mhs.net (S.D.); tergonzalez@mhs.net (T.G.); pferraro@mhs.net (P.F.); ahussein@mhs.net (A.H.);; 2 Memorial Health Care, Division of Internal Medicine, Pembroke Pines, FL 33028, USA; smuslehuddin@mhs.net; 3 Department of Pathology, University of Illinois at Chicago, Chicago, IL 60612, USA; mishadalal16@gmail.com

**Keywords:** bispecific antibodies, bispecific T cell engager, targeted therapy, personalized cancer therapy, cytokine release syndrome, precision medicine

## Abstract

Bispecific antibodies (BsAbs) have shown potential in cancer treatment and have become a rapidly growing field in cancer immunotherapy. Unlike monoclonal antibodies with two identical binding sites, BsAbs simultaneously bind two distinct epitopes on the same or different antigens, allowing for a range of mechanisms of action, including engaging immune cells to kill cancer cells and blocking signaling pathways. Despite regulatory approvals for hematological malignancies in the last decade, their clinical success in solid malignancies has been lacking until recently. There are currently five BsAbs approved by the FDA in the United States for solid tumors—amivantamab, tarlatamab, tebentafusp, zanidatamab and zenocutuzumab—and two BsAbs approved in China—cadonilimab and ivonescimab. Currently, several BsAbs are under clinical development for solid tumors, but are mostly in early phase I and II trials. This review provides an overview of the basic mechanism of action of BsAbs, current FDA-approved BsAbs, and current BsAbs under clinical development, their challenges in clinical use, the management of toxicities, and future directions.

## 1. Introduction

Bispecific antibodies (BsAbs) have shown promising efficacy in cancer treatment and have become a rapidly growing field in cancer immunotherapy. Unlike monoclonal antibodies with two identical binding sites, BsAbs simultaneously bind two distinct epitopes on the same or different antigens. The unique structure and possibilities of various designs allow for a range of mechanisms of action, including engaging immune cells to kill cancer cells and driving signaling pathway blockade [1]. Despite their regulatory approvals for hematological malignancies in the last decade, their clinical success in solid malignancies is lacking. There are seven BsAbs approved for hematological malignancies—blinatumomab, mosunetuzumab, glofitamab, epcoritamab, teclistamab, talquetamab, and elranatamab—and three BsAbs for non-cancer indications: emicizumab for hemophilia A, faricimab for neovascular age-related macular degeneration and diabetic macular edema, and ozoralizumab in Japan for rheumatoid arthritis [2,3,4,5]. The main reason for inadequate efficacy in solid malignancies could be due to the suppressive nature of the tumor microenvironment (TME), which inhibits T cell activity and promotes immune deficiency, the scarcity of tumor cell targets and their expression on normal cells, drug penetrance due to stromal barriers, and resistance due to tumor antigen loss [6].

There are currently five BsAbs approved by the Food and Drug Administration (FDA) in the United States for solid tumors: amivantamab for non-small-cell lung cancer (NSCLC), tarlatamab for extensive-stage small-cell lung cancer (ES-SCLC), tebentafusp for unresectable or metastatic uveal melanoma, zanidatamab for previously treated HER2-positive metastatic biliary tract cancer (BTC), and zenocutuzumab for NSCLC and pancreatic adenocarcinoma harboring an *NRG1* (neuregulin 1) gene fusion [7,8,9,10,11,12]. There are two BsAbs approved in China: cadonilimab for relapsed or metastatic cervical cancer and in combination with chemotherapy as a first-line treatment for advanced/metastatic gastric/gastroesophageal junction (GEJ) adenocarcinoma, and ivonescimab in combination with pemetrexed and carboplatin for patients with *EGFR*-mutated locally advanced or metastatic non-squamous NSCLC who have progressed after tyrosine kinase inhibitor (TKI) therapy [13,14,15]. Currently, several BsAbs are under clinical development for solid tumors, but are mostly in early phase I and II trials. This review provides an overview of the mechanism of action of BsAbs, current BsAbs under clinical development, challenges in clinical use, and future directions.

## 2. Mechanism of BsAbs

**Figure 1:**
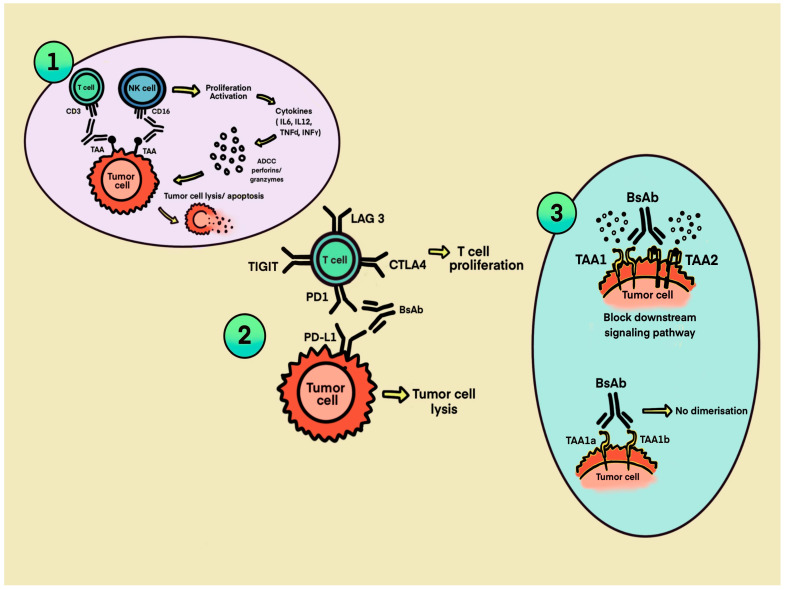
Mechanism of action of BsAbs: (1) Immune cell engagement—BsAbs can crosslink to either CD3 on T cells or CD16 on NK cells and TAA on tumor cells, which can lead to the activation of T or NK cells, causing the release of cytokines, perforins and granzyme B and lysis of tumor cells. (2) Immune checkpoint modulation—BsAbs can simultaneously bind to PD-L1 on tumor cells (or antigen-presenting cells) and either PD-1, CTLA-4, or LAG-3 on T cells, activating T cell response and the release of perforins and granzyme B and lysis of tumor cells. (3) Tumor-associated antigen blockade—BsAbs can simultaneously bind different targets TAA1 and TAA2 (such as EGFR and cMET receptors) on tumor cells and block phosphorylation and downstream signaling pathways or it can bind simultaneously to different domains on the same target TAA1a and TAA1b (such as two distinct domains of HER2 receptor), thus preventing dimerization and downstream signaling pathways.

The mechanism of BsAbs can be simply categorized into immune cell engagement, immune checkpoint blockade and tumor-associated antigen (TAA) blockade (Figure 1).

Immune cell engagement: Bispecific T cell engagers (BiTEs) are designed to bind to a TAA on cancer cells on one arm, and to receptors such as CD3 on T cells on another arm. This process causes T cell activation and immune response, which lead to tumor cell destruction [16]. This T cell activation leads to the secretion of cytokines and chemokines, which cause changes in the TME, and recruits more T cells and other immune cells [1]. On the other hand, bispecific killer cell engagers (BiKEs) bind to TAA on tumor cells and CD16 on natural killer (NK) cells, triggering cytokine release and antibody-dependent cellular toxicity (ADCC) [17].

Immune checkpoint blockade: BsAbs target immune checkpoints like PD-1 (programmed death receptor-1), CTLA-4 (cytotoxic T-lymphocyte associated protein 4), TIGIT (T cell immunoreceptor with Ig and ITIM domains), and LAG-3 (lymphocyte activation gene 3) on T cells on one arm, and on the other arm they bind to other immune checkpoint receptors on tumor cells, T cells or antigen-presenting cells [1]. This design could minimize on-target off-tumor toxicity while potentiating the antitumor immune response, thus reducing the compensatory upregulation of other immune checkpoint receptors seen with immune checkpoint-targeting monoclonal antibodies [1]. Some BsAbs are designed to bind to immune checkpoints on one arm and costimulatory receptors such as ICOS (inducible T cell costimulatory), OX40, and 4-1BB on the other arm, potentiating T cell response, while others bind to antigens in signaling pathways like VEGF (vascular endothelial growth factor) [1].

Tumor-associated antigen blockade: BsAbs bind to two distinct domains on a single target or two different targets in the signaling pathway, thereby enhancing the inhibition of downstream signaling pathways and cancer cell growth [1,6]. It can target two signaling pathways and induce internalization of receptors, leading to the prevention of receptor crosslinking and downstream signaling and the promotion of antibody-dependent cellular phagocytosis (ADCP) and ADCC by IgG-based BsAbs with Fc (fragment crystallizable) structure [1].

## 3. Current FDA-Approved BsAbs in Solid Tumors

Here, we will review five current FDA-approved BsAbs in solid tumors: tebentafusp, amivantamab, tarlatamab, zenocutuzumab and zanidatamab.

### 3.1. Tebentafusp (gp100 Targeting)

Glycoprotein 100 (Gp100) is a transmembrane glycoprotein which is expressed on normal melanocytes and melanoma cells and is involved in the maturation of melanosomes responsible for transporting melanin. Tebentafusp is a first-in-class T cell-engaging bispecific antibody (BsAb) that targets tumor cells that express gp100 presented by HLA*A02:01 and CD3 on T cells, redirecting T cells to destroy gp100-positive melanoma cells [18]. Its first-in-human phase I trial IMCgp-100-01 demonstrated promising efficacy with 16.7% partial responses (PRs) and 44.4% stable disease (SD) in 84 patients with cutaneous melanoma and 18 patients with uveal melanoma, and led to its phase III trial [19]. It received FDA approval for the treatment of unresectable or metastatic HLA-A*02:01-positive uveal melanoma in January 2022 [10]. The approval was based on a phase III IMCgp100-202 trial which evaluated previously untreated HLA-A*02:01-positive metastatic uveal melanoma patients [18]. The updated analysis reported a median overall survival (mOS) of 21.6 months in the tebentafusp group compared to 16.9 months in the control group (investigator’s choice of single-agent pembrolizumab, ipilimumab, or dacarbazine) (Hazard ratio HR 0.68; 95% CI: 0.54–0.87). Median progression-free survival (mPFS) was 3.4 months in the tebentafusp group and 2.9 months in the control group (HR: 0.76; 95% CI, 0.60–0.97) (see Table 1). The most common treatment-related adverse effects (TRAEs) were rash, pyrexia, pruritus, and hypotension, with rash being the most common grade 3 or 4 TRAE [18]. Tebentafusp was also evaluated in combination with durvalumab and/or tremelimumab in patients with metastatic cutaneous melanoma previously treated with a median of three prior lines in a phase I dose-escalation trial and it showed an objective response rate (ORR) of 14% with a tumor shrinkage rate of 41% and one-year survival rate of 76% [20]. It is currently being investigated in several clinical trials including a phase II trial of patients with HLA-A*02:01-positive previously untreated metastatic uveal melanoma (NCT06070012), patients with HLA-A*02:01-positive high-risk uveal melanoma following definitive treatment as adjuvant treatment (NCT06246149), and patients with large surgically unresectable primary uveal melanoma as a neoadjuvant treatment (NCT06414590) (see Table 2).

### 3.2. Amivantamab (EGFR-Targeting)

Amivantamab is a BsAb that targets both EGFR and cMET on tumor cells and it already has several FDA approvals. The design of amivantamab addresses the issue of emerging resistance to TKIs, caused by TKI-induced upregulation of alternative pathways such as the MET (mesenchymal–epithelial transition factor) pathway in EGFR-mutated NSCLC [1]. Amivantamab was initially granted accelerated approval by the FDA on 21 May 2021 for patients with advanced or metastatic NSCLC with *EGFR* exon 20 insertions who progressed on or after platinum-based chemotherapy [7]. The approval was based on a phase I multicenter, nonrandomized, multicohort CHRYSALIS trial in previously treated NSCLC patients with *EGFR* exon 20 insertions [7]. ORR was 40% with a median duration of response (mDOR) of 11.1 months and mPFS of 8.3 months [22]. The most common TRAEs included rash, infusion-related reactions and paronychia, which occurred in 86%, 66% and 45% of patients, respectively, whereas hypokalemia, rash, pulmonary embolism, diarrhea and neutropenia were the most common grade 3–4 TRAEs [22]. The recommended phase II dose (RP2D) was 1400 mg for patients greater than 80 kg and 1050 mg for patients less than 80 kg [22].

On 1 March 2024, amivantamab was approved by the FDA in combination with carboplatin and pemetrexed for the first-line treatment of locally advanced or metastatic NSCLC with *EGFR* exon 20 insertion mutations [7]. The approval was based on a phase III randomized, open-label multicenter PAPILLON trial which evaluated 308 NSCLC patients with *EGFR* exon 20 insertion mutations who had not received prior systemic therapy [30]. Patients were randomized 1:1 to receive amivantamab with carboplatin and pemetrexed or carboplatin and pemetrexed. The mPFS was 11.4 months in the amivantamab plus chemotherapy arm and 6.7 months in the chemotherapy-alone arm (HR: 0.40, 95% CI: 0.30, 0.53; *p*-value < 0.0001) [21]. ORR was seen in 73% and 47% of patients in the amivantamab–chemotherapy group and the chemotherapy group respectively (rate ratio, 1.50; 95% CI: 1.32 to 1.68; *p*-value < 0.001) [21]. The most common TRAEs were neutropenia (59%), paronychia (56%), and rash (54%) in the amivantamab–chemotherapy group and anemia (55%), neutropenia (45%), and nausea (42%) in the chemotherapy group [21]. Neutropenia is the most common grade 3 or higher TRAE in both groups [21].

The third FDA approval for amivantimab came on 20 August 2024 for the first-line treatment of patients with locally advanced or metastatic NSCLC with *EGFR* exon 19 deletions or exon 20 *L858R* mutations in combination with lazertinib [31]. The approval was based on a phase III MARIPOSA trial which randomized previously untreated patients with locally advanced or metastatic NSCLC with *EGFR* exon 19 deletions or *L858R* mutations into a 2:2:1 ratio of amivantamab–lazertinib, osimertinib, or lazertinib [23]. mPFS was significantly longer in the amivantamab–lazertinib group with 23.7 months compared to the osimertinib group with 16.6 months (HR: 0.70; 95% CI: 0.58 to 0.85; *p*-value < 0.001). ORR and OS at 24 months were 86% and 74% in the amivantamab–lazertinib group and 85% and 69% in the osimertinib group respectively [23]. 75% of the patients in the amivantamab–lazertinib group and 43% of patients in the osimertinib group had grade 3 or higher TRAEs, with paronychia and rash being the most common events [23]. Infusion-related reactions of amivantamab occurred mostly on cycle 1 day 1 of treatment and occurred in 63% of patients, while venous thromboembolic events occurred in 37% of patients in amivantamab-lazertinib group and in 9% of those in the osimertinib group [23]. At the recent European Lung Cancer Congress 2025 meeting, updated analysis showed that **amivantamab plus lazertinib** improved OS compared to osimertinib (HR for death: 0.75; 95% CI, 0.61–0.92; *p* < 0.005) at a median follow-up of 37.8 months. mOS was not estimable (NE; 95% CI, 42.9–NE) in the amivantamab-lazertinib arm compared to 36.7 months (95% CI, 33.4–41.0) in the osimertinib arm [24]. 

It might be challenging to use amivantamab due to its common infusion-related reactions, but a recent phase II SKIPPirr trial has shown that prophylaxis with 8 mg oral dexamethasone can effectively reduce amivantamab-related infusion reactions [32]. The most common TRAEs were dyspnea, hypotension and nausea [32]. In addition, subcutaneous formulation has also been developed to improve tolerability and reduce the administration time while maintaining efficacy [33]. However, it is not yet FDA-approved. Subcutaneous amivantamab was compared to intravenous amivantamab in combination with lazertinib in a phase III PALOMA 3 trial of refractory *EGFR*-mutated NSCLC patients and it showed similar efficacy with fewer adverse effects compared to intravenous amivantamab [33]. ORR was 30% in the subcutaneous and 33% in the intravenous group [33]. mPFS was 6.1 and 4.3 months, respectively, and OS was significantly longer in the subcutaneous versus intravenous group (HR: 0.62; 95% CI, 0.42 to 0.92; nominal *p* = 0.02) [33]. Fewer patients in the subcutaneous group experienced infusion-related reactions (13% vs. 66%) and venous thromboembolism (9% vs. 14%) in addition to reduced administration time of 4.8 min vs. 5 h compared to the intravenous group [33]. Given the high risk of venous thromboembolism, it is generally recommended to have primary thromboprophylaxis with either direct oral factor Xa inhibitor (apixaban and rivaroxaban) or low-molecular-weight heparin (LMWH) for the first 6 months of initiation of amivantamab and lizertinib if there is no contraindication with anticoagulation [34]. Amivantamab is currently being investigated in various clinical trials, as listed in Table 2.

### 3.3. Tarlatamab (DLL3-Targeting)

DLL3 (delta-like ligand 3) is an inhibitory ligand in the notch signaling pathway that is highly expressed on the surface of SCLC cells, making it a promising therapeutic target for SCLC. Tarlatamab (AMG757) is a first-in-class anti-DLL3 x CD3 bispecific T cell engager (BiTE) that binds to DLL3 on the surface of tumor cells and CD3 on cytotoxic T cells, leading to T cell activation and cytotoxic T cell-mediated cell death [9]. In a phase I study in patients with relapsed/refractory SCLC treated with tarlatamab, the ORR was 23.4% with an mDOR of 12.3 months [35]. The disease control rate (DCR) was 51.4% with a mPFS of 3.7 months and a mOS of 13.2 months [35]. Cytokine release syndrome (CRS) was the most common TRAE, occurring in 52% of patients, with 1% of patients having grade 3 CRS [35]. 

Tarlatamab received accelerated FDA approval for the treatment of patients with ES-SCLC who had disease progression after platinum-based chemotherapy [9]. The approval was based on a phase II DeLLphi-301 trial, where it was given every 2 weeks at a dose of 10 mg or 100 mg in patients with previously treated SCLC [25]. ORR and mPFS were 40% and 4.9 months in the tarlatamab 10 mg group and 32% and 3.9 months respectively in the 100 mg group [25]. OS at 9 months was 68% in patients in the 10 mg group and 66% in patients in the 100 mg group. CRS was the most common adverse event, occurring in 51% of the patients in the 10 mg group and in 61% of those in the 100 mg group. CRS occurred mainly during treatment cycle 1, with mostly grade 1 or 2 severity. Grade 3 CRS occurred less frequently in the 10 mg group (1%) compared to the 100 mg group (6%) [25]. The most common CRS symptoms were fever, hypotension and hypoxia. 8% of patients in the 10 mg group and 28% of patients in the 100 mg group had immune effector cell-associated neurotoxicity syndrome (ICANS) and associated neurologic events. Grade 3 or higher ICANs occurred in 5% of patients in the 100 mg group, but none were observed in the 10 mg group [25]. Its efficacy and safety are currently being confirmed in the phase III DeLLphi-304 trial in patients with SCLC who progressed on or after first-line platinum therapy [36].

It is currently being investigated in a phase III trial in combination with durvalumab compared to durvalumab alone in the first-line treatment of patients with ES-SCLC following treatment with platinum, etoposide and durvalumab (DeLLphi-305, NCT06211036) and a phase III randomized, placebo-controlled trial in patients with limited-stage SCLC (LS-SCLC) who have not progressed following concurrent chemoradiation (DeLLphi-306, NCT06117774) (Table 2).

### 3.4. Zenocutuzumab (HER2-Targeting)

Zenocutuzumab (MCLA128) is a BsAb that prevents human epidermal growth factor receptor HER2 and HER3 heterodimerization by using the “dock and lock” mechanism. It docks on HER2 and inhibits NRG1 binding to HER3 and also blocks the interaction of HER2 and HER3 [37]. NRG1 is a ligand that binds to HER3, which dimerizes with HER2 upon binding, and leads to downstream signaling activation. NRG1 fusion partners possess a transmembrane domain, which promotes cell growth by constitutively activating HER2/HER3 signaling and is found in 1% of solid tumors including lung, breast, pancreas, ovarian, and prostate [37].

The FDA granted accelerated approval to zenocutuzumab for advanced, unresectable or metastatic NSCLC and pancreatic adenocarcinoma harboring an *NRG1* gene fusion with disease progression on or after prior systemic therapy on 4 December 2024 [11]. The approval was based on an ongoing phase II eNRGy trial in patients with *NRG1* fusions [37]. The trial evaluated 65 patients with advanced or metastatic *NRG1* fusion-positive NSCLC and 27 adults with advanced or metastatic *NRG1* fusion-positive pancreatic adenocarcinoma who had disease progression following standard-of-care treatment [28,29]. It demonstrated an ORR of 34% with an mDOR of 12.9 months in previously treated patients with NSCLC and an ORR of 44% with an mDOR of 9.1 months in patients with pancreatic ductal adenocarcinoma with *NRG1* fusions [28,29]. The most common adverse reactions were diarrhea, musculoskeletal pain, fatigue, nausea, infusion-related reactions, dyspnea, rash, constipation, vomiting, abdominal pain, and edema [28,29]. It was also investigated in combination with trastuzumab and vinorelbine in a phase II trial of patients with HER2-positive metastatic breast cancer who progressed on prior anti-HER2 antibody drug conjugates with an ORR of 27% [38].

### 3.5. Zanidatamab (HER2-Targeting)

Zanidatamab is a BsAb that simultaneously binds to two distinct HER2 antigen-binding domains bound by trastuzumab and pertuzumab, thus promoting better binding and enhanced ADCC to kill cancer cells than tratuzumab and pertuzumab alone. The FDA granted accelerated approval to zanidatamab for previously treated, unresectable or metastatic HER2-positive (immunohistochemistry IHC3+) BTC patients on 20 November 2024 [12]. The approval was based on a phase IIb HERIZON-BTC-01 trial in which patients with HER2-amplified, unresectable, locally advanced or metastatic BTC who progressed on prior gemcitabine-based treatment were treated with zanidatamab 20 mg/kg intravenously every 2 weeks [12]. Cohort 1 has 80 patients with HER2-positive BTC (IHC 2+ or 3+) and cohort 2 has 7 patients with HER2-negative BTC (IHC 0 or 1+). Harding et al. reported results of cohort 1 which showed an ORR of 41.3% with an mOS of 15.5 months and an impressive mDOR of 14.9 months [26,27]. Diarrhea and infusion-related reactions were the most common TRAEs, which occurred in 32% and 33% of patients respectively [26]. Currently, its phase III trial in combination with cisplatin and gemcitabine with or without PD-1/PD-L1 inhibitors in the first-line setting for patients with metastatic BTC is ongoing (NCT06282575).

Zanidatamb also showed promising results in HER2-positive GEJ adenocarcinoma. Preliminary results of its combination with chemotherapy and tislelizumab (anti-PD-1 antibody) for the first-line treatment of HER2-positive GEJ adenocarcinoma in the phase Ib/II trial showed an ORR of 72.7% with 3% complete response (CR) and 69.7% PR and it is currently being investigated in a phase III HERIZON-GEA-01 trial [39,40]. It was also investigated as a neoadjuvant treatment in stage I node-negative HER2-positive breast cancer with 36% pathologic CR (pCR) [41]. However, it did not meet its primary endpoint in a phase II trial of previously treated HER2-positive metastatic endometrial carcinoma/carcinosarcoma, with only one patient achieving a PR [42]. It is currently being evaluated in combination with paclitaxel and ramucirumab in HER2-positive GEJ adenocarcinoma patients who failed at least one prior trastuzumab-containing regimen (NCT06043427).

## 4. Current BsAbs in Solid Tumors Under Investigation

### 4.1. HER2-Targeting BsAbs

HER2 is a transmembrane tyrosine kinase receptor, belonging to the epidermal growth factor receptor (EGFR) family, and plays an essential role in cell growth and survival [43]. Targeting HER2 has been rapidly evolving in various cancers, including breast, gastric, biliary tract, and lung cancers. HER2-targeting BsAbs are designed in different formats. Zanidatamab and KN026 are HER2 BsAbs designed to target two distinct domains of HER2, while zenocutuzumab targets both HER2 and HER3. Runimotamab (RG6194) is a BiTE that targets HER2 on tumor cells and CD3 on T cells, promoting T cell immune response. Zanidatamab and zenocutuzumab are the only two HER2-targeting BsAbs that received FDA approval as mentioned above.

Runimotamab (RG6194) is an HER2 x CD3 BiTE which is currently under a phase I trial as a single agent or in combination with trastuzumab in patients with metastatic HER2-positive breast cancer (NCT03448042).

KN026 is an HER2-targeted BsAb that binds two distinct domains, II and IV, of HER2, that has been tested in early-phase clinical trials in both metastatic and neoadjuvant settings. In a study by Xu et al., KN026 showed an ORR of 56% with an mDOR of 9.7 months in 27 patients with advanced HER2-expressing gastric or GEJ cancer who received at least one prior line of treatment [44]. It demonstrated an ORR of 28.1% with an mPFS of 6.8 months in 57 female patients with HER2-positive metastatic breast cancer who had progressed on prior HER2 therapies. The most common TRAEs are pyrexia, diarrhea, and increased transaminases [45]. KN026 in combination with docetaxel as a neoadjuvant treatment in HER2-positive early breast cancer in a phase II trial showed a pCR rate of 50% [46]. It is currently investigated in a phase II trial of patients with HER2-positive CRC and BTC (NCT05985707) and a phase III trial in combination with HB1801 as a neoadjuvant treatment for early or locally advanced HER2-positive breast cancer (NCT06747338).

YH32367 (ABL105) is an HER2 x 4-1BB targeting BsAb. 4-1BB or CD137, part of the tumor necrosis factor receptor superfamily, is a costimulatory receptor expressed on activated T cells, NK cells, and other immune cells and it binds to its ligand 4-1BBL on antigen-presenting cells, facilitating cytotoxic immune activation [47]. It has shown significant long-term antitumor efficacy in h4-1BB KI mice with HER2-low and HER2-positive tumors and has synergic activity with anti-PD-1 antibody in h4-1BB KI mice bearing HER2-low tumors [48]. There is currently a phase I dose-escalation study in patients with HER2-positive locally advanced or metastatic solid tumors (NCT05523947) and a dose-optimization study for the RP2D [48].

Izalontamab (SI-B001) is a bispecific IgG antibody that targets both EGFR and HER3 receptors, thus preventing both EGFR and HER3-mediated signaling pathways. Its combination with docetaxel in cohort B of patients with locally advanced or metastatic *EGFR* and *ALK* wild-type NSCLC who failed first-line treatment showed an ORR of 45.5% [49]. Izalontamab with paclitaxel in patients with recurrent and metastatic HNSCC progressed on prior anti-PD-1/L1 with platinum-based chemotherapy without prior exposure to paclitaxel showed an ORR of 64.3% [50]. It is currently under several clinical trials, including on metastatic NSCLC and HNSCC in China (NCT05943795, NCT06668961, NCT05054439) (see Table 2).

### 4.2. PSMA Targeting BsAbs

Prostate-specific membrane antigen (PSMA), also known as glutamate carboxypeptidase II, is a type II transmembrane protein associated with folate hydrolase activity, which is primarily produced by cells in the prostatic epithelium [51]. It is highly expressed in androgen-independent prostate cancer and limitedly expressed in non-prostatic tissues, making it a desirable target for prostate cancer treatment [51]. However, there are a lot of challenges with PSMA BsAbs in the clinical setting due to its duration of responses and adverse effects, and no PSMA BsAbs are currently approved by the FDA.

Pasotuxizumab (AMG 212 or BAY 2010112) is a first-in-human anti-PSMA x CD3 BiTE that was evaluated in the phase I dose-escalation study in patients with metastatic castration-resistant prostate cancer (mCRPC) [52]. Despite its efficacy of lowering PSA, subcutaneous dosing is not feasible due to the development of neutralizing endogenous anti-drug antibodies, leading to non-sustained and mitigated responses. Production of anti-drug antibodies was resolved by continuous intravenous treatment, but the administration was too complicated to justify further development of the drug [53].

Acapatamab (AMG 160) is a second-generation anti-PSMA x CD3 BiTE with an additional Fc fragment linked to the BiTE molecule core, thus extending its half-life, with endogenous transcytosis and recycling mechanisms [54]. In a phase I study of acapatamab in mCRPC refractory to hormonal therapy and taxane-based chemotherapy, PSA reductions were seen in 30.4% of patients and radiographic PRs were seen in 7.4% of patients [55]. CRS was the most common TRAE, which mostly occurred in cycle 1. Grade 3 or higher CRS was observed in 23.4% and 16.1% in dose-exploration and dose-expansion studies, respectively. Treatment-emergent anti-drug antibodies were detected in 55% of patients [55]. It was discontinued by the manufacturer and no further developments were made.

AMG 340 is a novel PSMA x CD3 BiTE with a low-affinity anti-CD3 binding domain to mitigate off-target immune activation and CRS. However, its phase I trial in patients with mCRPC who had received more than two prior lines of treatment showed no PR or CR (14 patients with SD) despite its low CRS toxicities [56]. The most common TRAEs were CRS (52%; grade 1, 26%; grade 2, 24%; grade ≥ 3, 2%) and thrombocytopenia (24%; grade 2, 10%; grade ≥ 3, 14%) [56]. Further developments of AMG 340 have been halted.

JNJ-081/ JNJ63898081 is another anti-PSMA x CD3 BiTE that targets PSMA-expressing tumor cells and CD3-expressing T cells. Preliminary results of its phase I dose-escalation study in previously treated patients with mCRPC showed transient PSMA decline with subcutaneous injection without radiographic responses [57]. Grade 2 CRS was observed at higher doses but was decreased by subcutaneous injection and step-up dosing [57]. It also causes anti-drug antibodies in 19 patients, thus prompting further investigation of next-generation BsAbs.

REGN5678 is an anti-PSMA x CD28 BiTE which showed antitumor activity when combined with cemiplimab in a phase I dose-escalation study, indicating potential to overcome mCRPC resistance to PD-1 inhibition. Radiographic response per RECIST 1.1 occurred in one in three of patients at 30 mg (CR), one in four at 100 mg (PR), and all patients at 300 mg doses (PR) [58]. CRS occurred in 17% of patients [58]. It is currently being investigated in combination with cemiplimab in patients with mCRPC and other tumors (NCT03972657).

### 4.3. Claudin 18.2-Targeting BsAbs

Claudin18.2 (CLDN18.2) is highly expressed on gastric mucosa epithelial cells and other malignant tumors including lung, esophageal, and pancreatic cells. Zolbetuximab is currently the first and only FDA-approved drug targeting Claudin 18.2 but it is a mouse chimeric monoclonal antibody with human IgG1 designed to bind CLDN18.2 [59]. The approval was based on the phase III GLOW and SPOTLIGHT trials where zolbetuximab was combined with chemotherapy (FOLFOX or CAPOX—capecitabine and oxaliplatin) for advanced, unresectable or metastatic gastric/GEJ adenocarcinoma patients with CLDN18.2 positivity (≥75% of tumor cells with moderate-to-strong CLDN18.2 membranous staining) [59]. Currently, there are no claudin 18.2-targeting BsAbs approved yet, but several of them are currently under investigation [59].

Gresonitamab (AMG 910) is a half-life-extended CLDN18.2 x CD3 BiTE. Its phase I trial enrolled patients with relapsed or refractory metastatic or locally advanced unresectable CLDN18.2-positive gastric/GEJ adenocarcinoma following two prior lines of therapy but was terminated as of April 2024 [60]. 

AZD5863, another CLDN18.2 x CD3 BiTE with bivalent high-affinity binding to CLDN18.2 and low-affinity binding to CD3, has shown efficacy in preclinical in vitro and vivo studies [61]. It is currently being investigated in a phase I/II trial for patients with advanced or metastatic solid tumors, including gastric, pancreatic, and esophageal adenocarcinoma (NCT06005493) [62].

LNF2007 is another CLDN18.2 x CD3 BiTE with a relatively weak but decent affinity to CD3 to minimize off-target toxicity and CRS and maximize therapeutic efficacy [63]. It demonstrated similar efficacy to AMG 910 and AZD5863 but was safer in preclinical studies [63]. It is currently evaluated in a phase I dose-escalation and dose-expansion study in CLDN18.2-positive advanced solid tumors (preferably gastric, pancreatic cancer and BTC) in China (NCT06752447).

ASP2138 is an asymmetric 2 + 1 BiTE, comprising a bivalent humanized anti-CLDN18.2 antigen-binding domain and a monovalent anti-CD3 domain, that is currently being investigated alone or in combination with standard treatment (mFOLFIRINOX—fluorouracil, irinotecan, oxaliplatin; mFOLFOX6 or ramucirumab and paclitaxel) in a phase I/II trial of patients with CLDN18.2-positive advanced gastric/GEJ or pancreatic cancer (NCT05365581) [64].

Q-1802 is a BsAb that targets both CLDN18.2 and PD-L1, causing ADCC against tumor cells by targeting CLDN18.2, while activating innate and adaptive immunity by blocking the anti-PD-L1 portion and subsequent PD-1. Its phase II clinical trial is currently enrolling patients with CLDN18.2-expressing advanced or metastatic solid tumors in China [65]. Preliminary results showed 70% PR and 30% SD in the 10 mg/kg group and 50% PR and SD each in the 20 mg/kg group (NCT04856150) [65]. Currently, a phase I/II trial of Q1802 in combination with XELOX is underway in patients with advanced or recurrent metastatic CLDN18.2-positive primary gastric/GEJ adenocarcinoma in China (NCT05964543).

### 4.4. CEA-Targeting BsAbs

Carcinoembryonic antigen (CEA), a glycoprotein involved in cell adhesion, is a valuable tumor marker as its level is significantly increased in certain types of cancer, including colorectal cancer (CRC), while its level is low in healthy individuals [66]. High levels of CEA can also be found in breast, lung, ovarian, gastric, and pancreatic cancers [66]. It seems like a reasonable target for cancer treatment but there have not been any promising results in BsAbs targeting CEA.

MEDI565 (AMG211), a CEA x CD3 BiTE, has shown some antitumor activity by promoting T-cell-mediated tumor cell lysis regardless of *KRAS*, *BRAF*, *PIK3CA* or *TP53* mutations in preclinical studies, but the phase I dose-escalation study of patients with gastrointestinal adenocarcinomas showed no objective responses with only 28% SD [67]. It was discontinued by the parent company in 2019.

Cibisatamab (RO6958688) is a CEA x CD3 BiTE with an asymmetric Fc structure, with one arm harboring a CEA-binding Fab (fragment antigen binding antibody) and the other arm consisting of a CD3-binding Fab in a distinct “head to tail” configuration. It was investigated in a phase I trial of advanced or metastatic CEA-positive solid tumors with or without obinutuzumab in S1 (NCT02324257) and with atezolizumab in S2 (NCT02650713) [68]. It showed 4% ORR in S1 and 7% ORR in S2, while patients with microsatellite stable CRC had an ORR of 14% in S2 [68]. Anti-drug antibodies (ADAs) were seen in 40% of patients in S1 and 52% of patients in S2; therefore, ADA occurrence seemed to have been mitigated by pretreatment with Obinutuzumab. The most common TRAEs were pyrexia, infusion-related reactions, and diarrhea [68]. Further research and investigations are currently being conducted to improve the design and geometry of CEA-targeting BiTEs [69].

### 4.5. EpCAM-Targeting BsAbs

Epithelial cell adhesion molecule (EpCAM), a transmembrane glycoprotein, plays an essential role in the regulation of intramembrane proteolysis-mediated signaling and activation of Wnt signaling, promoting tumor cell growth and migration. Therefore, it is considered an attractive target in cancer treatment [70]. Its overexpression is found in colorectal, gastric, breast, prostate, and lung cancers [71]. However, targeting EpCAM can be challenging as it is also found in normal epithelial tissues, thus limiting its utility due to potential adverse effects on normal cells [71]. This led to the development of a conditionally active biological (CAB) BsAb that binds to both EpCAM and CD3 in an acidic tumor environment but limits binding to EpCAM and CD3 in normal tissues [71].

Catumaxomab is a rat/murine-hybrid trifunctional monoclonal BiTE targeting EpCAM x CD3, which was approved back in April 2009 as the first BsAb in the European Union for the treatment for patients with malignant ascites and EpCAM-positive carcinomas [72]. It has been investigated in several clinical trials including a phase II trial in combination with chemotherapy in gastric cancer patients with peritoneal carcinomatosis [73]. In another phase II/III trial, 258 patients with malignant ascites due to epithelial cancer were randomized to paracentesis plus catumaxomab vs. paracentesis alone [74]. Puncture-free survival was significantly longer at 46 days in the catumaxomab group compared to 11 days in the paracentesis-alone group (HR 0.254, *p* < 0.0001); the median time to next paracentesis was 77 days versus 13 days (*p* < 0.0001) [74]. However, it is no longer being manufactured due to logistic considerations [75].

Solitomab (AMG 110) is another EpCAM x CD3 BiTE which has shown immune-mediated cytotoxic activity in preclinical studies [76]. However, in a multicenter phase I trial in advanced refractory solid tumors, solitomab was associated with dose-limiting toxicities including severe diarrhea and increased liver enzymes, which halted dose escalation to potential therapeutic levels [77].

BA3182 is another EpCAM x CD3 BiTE, and its phase I dose-escalation study is currently enrolling patients with advanced adenocarcinoma (NCT05808634). M701 is an EpCAM x CD3 BiTE that is currently being evaluated for the treatment of malignant ascites associated with advanced epithelial solid tumors in patients who have received at least two prior systemic therapies (NCT06432296), and preliminary results of M701 with systemic therapy of the investigator’s choice showed longer puncture-free survival and OS compared to patients with systemic therapy alone [78]. It is also currently being evaluated in a phase I trial of patients with advanced adenocarcinoma (NCT05808634).

BNT314 (GEN1059) is another BsAb that targets EpCAM and the costimulatory receptor 4-1BB. It is currently being evaluated in combination with pembrolizumab in a phase I/II trial of previously treated patients with metastatic or advanced malignant solid tumors (NCT06150183) [79].

### 4.6. GPC3-Targeting BsAbs

Glyican-3 (GPC3) is a GPI-anchored cell surface oncofetal proteoglycan which is overly expressed in fetal liver and hepatocellular carcinoma (HCC) but not in normal liver. Therefore, it is a potential target for the treatment of HCC [80,81]. GPC3 is presumed to cause HCC progression by interacting with growth factors and activating the Wnt signaling pathway [81].

ERY974 is a humanized IgG4 BiTE targeting GPC3 and CD3. It showed significant non-immunogenic antitumor effects in tumors that were unresponsive to treatment with immune checkpoint inhibitors (ICIs) as well as demonstrated synergistic activity with chemotherapy in non-inflamed tumors in xenograft models [82]. It was evaluated in early phase I trials in patients with advanced solid tumors with minimal response and dose limiting due to CRS [83,84]. It is currently being investigated in an early phase I dose-escalation study in patients with locally advanced or metastatic HCC (NCT05022927). SAR444200 is another GPC3 x CD3 BiTE which is under investigation in a phase I/II trial of advanced solid tumors (NCT05450562).

### 4.7. HLA-G-Targeting BsAbs

Human leukocyte antigen G (HLA-G) is non-classical major histocompatibility class I (MHC 1) molecule at the maternal–fetal interface with minimal expression on normal tissues, and it plays an essential role in organ transplantation, viral infections, autoimmunity and oncogenesis [85]. It is highly expressed in solid tumors including renal cell carcinoma (RCC, 75%) and ovarian (61%), colon (64%) and rectal cancers (40%) and moderately expressed in lung adenocarcinoma, endometrial, and pancreatic cancers [85].

JNJ-78306358 is a first-in-class HLA-G x CD3 BiTE that binds with weaker affinity to CD3 expressed on T cells and higher affinity to HLA-G-expressing tumor cells [85]. Despite its activity in preclinical studies, a phase I dose-escalation study of patients with metastatic or unresectable solid tumors with high expression of HLA-G was limited, with adverse effects including CRS, increased transaminases, and pneumonitis [85,86].

### 4.8. Immune Checkpoint-Targeting BsAbs

#### 4.8.1. PD-1/CTLA-4-Targeting BsAbs

PD-1 and CTLA-4 on immune cells are counterreceptors for the B7 family of costimulatory molecules and are the negative regulators of T cell activation. PD-1 and CTLA-4 act as a break on T cells and, upon binding to their ligands, they prevent T cells from attacking other cells [87]. Therefore, immune checkpoint blockade activates the immune system to attack cancer cells by utilizing therapeutic antibodies to block regulatory checkpoints that inhibit immune responses. There have been several PD-1/PD-L1 and CTLA-4 monoclonal antibodies approved for cancer treatment; thus, designing BsAbs using biochemical engineering to target different immune checkpoints seems to provide a promising effective treatment strategy in cancer therapy [87]. A front-runner amongst PD-1 x CTLA-4 BsAbs is cadonilimab, which is currently approved in China but not in the United States [14].

Cadonilimab (AK104) is a BsAb that targets both PD-1 and CTLA-4 immune checkpoint receptors that is currently approved in China for the treatment of relapsed or metastatic cervical cancer [15]. In an early phase Ib/II trial of patients with advanced solid tumors, it showed ORRs of 32.3% in the cervical cancer cohort, 18.2% in the esophageal squamous cell carcinoma cohort, and 16.7% in the HCC cohort [88]. The approval was based on the phase III COMPASSION 16 trial in combination with first-line platinum-based chemotherapy with or without bevacizumab in 445 patients with persistent, recurrent or metastatic cervical cancer in China [89]. The mPFS was 12.7 months and mOS was not reached in the cadonilimab group, while the mPFS was 8.1 months and the mOS was 22.8 months (95% CI: 17.5–29.0, HR: 0.64, *p* = 0.0011) in the placebo group [89]. It was also recently approved in China in combination with fluoropyridine and platinum-based chemotherapy for the first-line treatment of patients with locally advanced, unresectable or metastatic gastric/GEJ adenocarcinoma [15]. The approval was based on the COMPASSION-15 trial, which investigates cadonilimab in untreated, HER2-negative, locally advanced, or metastatic gastric or GEJ cancer, including those with PD-L1-low tumors, compared with chemotherapy alone [15]. The cadonilimab plus chemotherapy group has an mOS of 15.0 months compared to 10.8 months in the placebo plus chemotherapy group (HR, 0.62; 95% CI, 0.50–0.78; *p* < 0.001) [15,90]. It is currently under investigation in several clinical trials including various types of cancers including NSCLC, neuroendocrine, pancreatic cancers, HCC, triple-negative breast cancers, nasopharyngeal cancers, muscle-invasive bladder cancers, melanoma, and endometrial cancers in China (Table 2).

Volrustomig (MEDI5752) is a novel monovalent PD-1 x CTLA-4 BsAb. It showed promising efficacy in combination with chemotherapy compared to pembrolizumab plus chemotherapy as a first-line treatment in NSCLC with an improved ORR of 55.6% vs. 30.0% and mPFS of 13.4 months vs. 9.0 months; mOS was not reached vs. 16.5 months in the PD-L1 < 1% group [91]. It is currently undergoing a phase III clinical trial in combination with carboplatin and pemetrexed in unresectable pleural mesothelioma compared to platinum plus pemetrexed or nivolumab plus ipilimumab (eVOLVE-Meso) (NCT06097728) [92]. It is also being compared to placebo after definitive chemoradiation in the phase III eVOLVE-Cervical trial in patients with high-risk locally advanced cervical cancer (FIGO stage IIIC to IVA) (NCT06079671) [93]. It is currently being investigated in several trials including those listed in Table 2.

Lorigerlimab (MGD019) is a tetravalent bispecific PD-1 x CTLA4 dual-affinity re-targeting (DART) molecule with increased activity on dual PD-1/CTLA-4-expressing cells, and it has been shown to have T cell responses to the levels obtained by nivolumab and ipilimumab [94]. Its early phase I dose-escalation trials in patients with mCRPC and advanced solid tumors have demonstrated some antitumor activity, with an ORR of 25.7% with a manageable safety profile [94,95]. It is currently in several phase II trials, as listed in Table 2.

Erfonrilimab (KN046) is an anti-PD-L1 x CTLA-4 BsAb that was investigated in a phase II trial in combination with nab-paclitaxel as a first-line treatment in patients with metastatic triple-negative breast cancer [96]. It showed an ORR of 44%, mPFS of 7.33 months, mOS of 30.92 months and mPFS of 8.61 months [96]. The most common TRAEs were neutropenia, leukopenia, alopecia, elevated alanine aminotransaminase (ALT) and elevated aspartate aminotransferase (AST) [96]. It has also demonstrated promising antitumor activity in a phase I trial of advanced solid tumors with ORR of 12.5% and mDOR of 16.6 months [97]. Its phase II study in patients with NSCLC who failed platinum-based therapy showed promising efficacy with both 3 mg/kg and 5 mg/kg dosing [98]. It is also currently being investigated in several phase I/II trials in patients with advanced solid tumors in China, as listed in Table 2.

Vudalimab (XmAb20717) is another PD-L1 x CTLA-4 BsAb that showed an ORR of 14.1% in a phase I dose-expansion study of patients with advanced solid tumors including patients who progressed on prior ICI [99]. A phase I dose-escalation study of patients with selected advanced solid tumors demonstrated one CR in a melanoma patient who progressed on prior pembrolizumab at 10 mg/kg (highest dose level). The most common grade 3 or 4 TRAEs were the elevation of transaminases, rash, lipase, and amylase [100]. It is currently being investigated in a phase II clinical trial either alone or in combination with chemotherapy or targeted therapy in patients with mCRPC who progressed on prior therapy (NCT05005728) and a phase I/II trial in combination with chemotherapy as a first-line treatment in advanced NSCLC (NCT06173505).

#### 4.8.2. PD-1/TIGIT-Targeting BsAbs

T cell immunoreceptor with immunoglobulin (Ig) and ITIM domain (TIGIT) is a receptor of the Ig superfamily, which is expressed by activated T cells, NK cells, regulatory T cells and follicular T helper cells and limits T cell- and NK cell-mediated tumor recognition. TIGIT binds to two ligands, CD155 and CD112, that are expressed by tumor cells and antigen-presenting cells in the TME. In tumor cells, TIGIT is co-expressed with PD-1 on tumor antigen-specific T cells and tumor-infiltrating lymphocytes [101].

Rilvegostomig (AZD2936) is a monovalent humanized IgG1 anti-PD-1 x TIGIT BsAb that has shown potential antitumor activity in preclinical and early phase I/II trial of advanced/metastatic NSCLC [102,103]. It is currently being evaluated in the global phase III trial ARTEMIDE-Lung03 of rilvegostomig or pembrolizumab in combination with platinum-based chemotherapy for the first-line treatment of patients with metastatic NSCLC whose tumors express PD-L1 (≥1%) (NCT06627647).

#### 4.8.3. PD-1/IL2-Targeting BsAbs

Interleukin-2 (IL-2) is a cytokine produced mainly by activated CD4+ T cells that plays an important role in the proliferation, survival, and function of immune effector cells, regulatory T cells and NK cells [104].

IBI363 is a PD-1 x IL-2 BsAb that binds to both PD-1 expressed on PD-1-expressing T cells and IL-2 receptor (IL2R). This process promotes the induction of certain cytotoxic cytokines, such as interferon-gamma (IFNg) and transforming growth factor-beta (TGF-β), leading to enhanced T cell-mediated immune response against tumor cells [104]. By selectively activating PD-1 positive T cells by IL-2, but not PD-1-negative bystander or naive T cells, it reduces IL2-induced systemic toxicity [105]. It has shown promising antitumor activity in patients with advanced melanoma in its first-in-class phase I trial [105]. The trial enrolled 17 patients with cutaneous melanoma, 22 patients with acral melanoma, 25 patients with mucosal melanoma and 3 patients with unknown primary melanoma. ORR was 28.1% in patients without prior immunotherapy and 21.2% in patients who had prior immunotherapy [105]. The most common adverse events were arthralgia, hyperthyroidism, and anemia [105]. Its phase I trial (NCT05460767) presented at the 2024 IASLC World Conference on Lung Cancer showed an ORR of 50% and DCR of 88.9% in patients with squamous NSCLC who had received prior immunotherapy [106]. It has received fast-track designation from the United States FDA as a monotherapy for advanced melanoma and metastatic NSCLC [107]. There are several ongoing phase I/II trials of IBI363 in advanced solid tumors, including gastric cancer, melanoma, CRC, and NSCLC, in China and the United States (Table 2).

#### 4.8.4. PD-1/ICOS-Targeting BsAb

XmAb23104 is a novel BsAb that targets PD1 and ICOS, which is a co-stimulatory molecule which is highly expressed by activated CD4+ and CD8+ T cells [108]. A total of 62 patients with advanced solid tumors were enrolled in a phase I dose-escalation study; 59.7% experienced TRAEs, and the most common TRAEs were diarrhea, decreased appetite and fatigue [108]. In the dose-escalation part of the study, XmAb23104 was generally well tolerated at doses up to 15 mg/kg, and a dose of 10 mg/kg was selected after consideration of pharmacokinetics, clinical safety and activity data [108]. It was investigated in combination with XmAb22841 (CTLA-4 x LAG-3) in patients with metastatic melanoma refractory to prior ICI, but the trial was ultimately terminated [109].

#### 4.8.5. PD-1/LAG3-Targeting BsAbs

Lymphocyte-activation gene 3 (LAG-3) is a protein functioning as an immune checkpoint receptor. The PD-1 x LAG-3 BsAb targets both PD-L1 and LAG-3, enhancing effector CD4+ and CD8+ T cell activation [110].

Tebotelimab (MGD013) is a PD-1 × LAG-3 BsAb that was evaluated in an early phase I trial in patients with solid tumors [110]. It demonstrated a dismal ORR of 3.3% in a phase I/II dose-escalation trial in patients with advanced HCC who had failed prior targeted therapy [111]. It showed an ORR of 24% with a DCR of 40% in untreated, unresectable, recurrent or metastatic mucosal melanoma in early phase I study [112]. Currently, there are no active clinical trials for tebotelimab.

#### 4.8.6. PD-L1/PD-1 BsAbs

LY3434172 (IBI318) is a first-in-class PD-L1 x PD-1 BsAb that has shown preliminary efficacy in early phase I/II trials of advanced tumors. It showed ORRs of 45.5% in treatment-naive NSCLC patients and 30.0% in immunotherapy-naive nasopharyngeal cancer patients who failed platinum-based treatment [113]. It is currently being investigated in combination with lenvatinib in a phase I trial in patients with advanced NSCLC in China (NCT04777084).

CTX8371 is a novel tetravalent PD-L1 x PD-1 BsAb that is currently in a phase I dose-escalation trial in advanced malignancies, including NSCLC, triple-negative breast cancer, Hodgkin’s Lymphoma, HNSCC, and malignant melanomas (NCT06150664) [114].

#### 4.8.7. PD-L1/TIM-3-Targeting BsAbs

T-cell immunoglobulin and mucin domain 3 (TIM3), a transmembrane protein and immune checkpoint receptor, is expressed on T cells, NK cells, and tumor-infiltrating lymphocytes (TILs) and is co-expressed with PD-1 on tumor antigen-specific T cells [115]. TIM-3 binds to four distinct ligands including Galectin-9, PtdSer, CEACAM1, and HMGB1 and could inhibit the Th1 (type I T helper cell) response by inducing apoptosis [116,117]. Thus, co-targeting PD-L1 and TIM3 can promote antitumor activity in tumors with a high expression of TIM-3 [116].

LY3415244 is a PD-L1 x TIM-3 BsAb. A phase I trial was conducted in patients with advanced tumors with doses escalating from 3 mg to 70 mg every 2 weeks. However, it was terminated during the dose-escalation phase due to unexpected immunogenicity as all patients developed treatment-emergent anti-drug antibodies (NCT03752177) [118].

#### 4.8.8. PD-L1/CD47-Targeting BsAbs

CD47 is a transmembrane glycoprotein of the Ig superfamily, which is a potent “do not eat me” signal. CD47 interaction with signal regulatory protein alpha (SIRPalpha) inhibits macrophage activation and protects cancer cells from phagocytosis and T cell activation [119].

IBI322 is an anti-CD47 x PD-L1 BsAb that is designed based on the “knobs-into-holes” strategy and blocks both PD-1/PD-L1 and CD47/ SIRPalpha pathways [120]. By targeting both CD47 and PD-L1 with a higher binding affinity to PD-L1, IBI322 more selectively blocks CD47 on tumor cells expressing both CD47 and PD-L1 than tumors expressing CD47 alone, thus minimizing adverse effects caused by the blockade of CD47 expressed on healthy red blood cells [120]. An early phase I study of IBI322 in 58 patients with advanced solid tumors showed some antitumor activity (20% PR and 35% SD) in China [120]. The most common TRAEs were anemia, thrombocytopenia, and pyrexia, with the most common grade ≥3 TRAE being thrombocytopenia [120]. It has also shown promising activity in the phase I trial of 24 patients with anti-PD-1 or PD-L1 treatment-resistant classical Hodgkin lymphoma with an ORR of 47.8% and DCR of 91.3% [121]. It is currently being investigated in a phase II trial in combination with lenvatinib in ES-SCLC patients who failed first-line PD-L1 inhibitors in China (NCT05296603).

#### 4.8.9. PD-L1/4-1BB-Targeting BsAbs

FS222 is a tetravalent PD-L1 x 4-1BB BsAb, and its first-in-human phase I trial in advanced solid tumors, including PD-1 refractory melanoma, showed promising antitumor activity, including 60% ORR in post-PD-1-treated metastatic cutaneous melanoma [122]. Its phase I trial is currently recruiting previously treated patients with advanced tumors (NCT04740424).

There are other novel PD-L1 x 4-1BB BsAbs such as MCLA145, ATG101 (NCT04986865) and ABL503 (NCT04762641), which have been evaluated in early phase I/II trials in advanced solid tumors [123,124].

In a phase I study of MCLA145 either alone or in combination with pembrolizumab in patients with cancers that are either immunotherapy-naive or relapsed on prior immunotherapy, 72 patients with 26 cancer types were enrolled. In 52 patients treated with MCLA145 monotherapy, there were five PRs in patients with glioblastoma, sarcoma, and cervical, anal, and gastric cancer. In the combination arm with 19 evaluable patients, 1 patient with Merkel cell carcinoma had PR and 1 patient with PD-L1-positive NSCLC had CR. DCR was seen in 37% with monotherapy and 68% with combination therapy [124].

A phase I trial of ABL503 in patients with advanced or relapsed/refractory solid tumors showed one CR in a patient with ovarian cancer who received six prior lines of treatments and five PRs in patients with ovarian, melanoma, gastric, HNSCC and esophageal cancer. Thus, ABL503 is a potential treatment for patients with different cancer types who have progressed on several lines of treatment [123]. Currently, it is being investigated in a phase I dose-escalation and -expansion study in patients with advanced or metastatic solid tumors (NCT04762641).

#### 4.8.10. PD-L1/OX40-Targeting BsAbs

OX40 (CD134) is a transmembrane receptor that express on T cells, NK cells, and neutrophils, while its ligand OX40L is expressed on activated antigen-presenting cells such as dendritic cells, B cells and macrophages. OX40 promotes cell proliferation, suppresses apoptosis and enhances T cell activation; however, T cells must first be activated to express OX40 [116].

KN051 is a PD-L1 x OX40 BsAb that has shown antitumor activity in preclinical studies, but its phase I study (NCT05309512) in China has been terminated due to the sponsor’s clinical development strategy adjustment [125]. Another PD-L1 x OX40 BsAb, EMB-09, also demonstrated antitumor activity in preclinical studies and is currently in a phase I trial of advanced or metastatic solid tumors (NCT05263180) [126].

### 4.9. EGFR-Targeting BsAbs

#### 4.9.1. EGFR/cMET Targeting BsAbs

EGFR and cMET activate intracellular signal transduction, promoting cancer cell proliferation and survival. Resistance to TKIs is associated with increased cMET signaling in *EGFR*-mutant NSCLC [127]. Amivantamab is an EGFR x cMET BsAb which is the first and only drug in this category to have FDA approval as mentioned above.

Bafisontamab (EMB01) is a novel EGFR x cMET BsAb. Preliminary data of its phase I/II trial indicated its activity in *EGFR*-driven advanced or metastatic NSCLC, including patients with acquired EGFR TKI resistance and *EGFR* exon 20 insertions [127]. Two patients had PR; fourteen patients had SD with a DCR of 42.1% [127]. It is currently being investigated in early phase I/II trials in combination with osimertinib in patients with *EGFR*-mutant NSCLC who progressed on standard treatment (NCT05498389), in advanced or metastatic gastrointestinal cancer (NCT05176665), and in previously treated *EGFR-* and/or *cMET*-mutated advanced or metastatic solid tumors (NCT03797391) in the United States and China (see Table 2).

MCLA-129 is another EGFR x cMET BsAb [128]. Its first-in-human phase I/II study evaluated MCLA-129 monotherapy in advanced/metastatic NSCLC patients in different cohorts, including NSCLC patients with a *c-MET* exon 14-skipping mutation in cohort A, cohort B with *EGFR* exon 20 insertion mutation, and cohort C with sensitized *EGFR* mutation. ORRs in cohorts A, B and C were 43.5%, 28.6% and 21.8%, respectively, and patients in cohort A who received prior MET TKI had an ORR of 37.5% [129]. It is currently being investigated either alone or in combination with osimertinib or chemotherapy in a phase I/II trial of patients with advanced NSCLC and other solid tumors with or without *EGFR*, *cMET* exon 14-skipping mutation or *EGFR* exon 20 insertion (NCT04868877).

#### 4.9.2. EGFR/LGR5-Targeting BsAbs

Leucine-rich repeat-containing G-protein coupled receptor 5 (LGR5), a member of the Wnt signaling pathway, is a transmembrane receptor that is highly expressed on certain cancer stem cells and plays an essential role in tumor cell proliferation and survival [130].

Petosemtamab (MCLA158) is a IgG1 EGFR x LGR5 BsAb that targets and binds to both EGFR and LGR5, thus inhibiting the activation of both EGFR- and LGR5-mediated signaling pathways [130]. Its phase I/II trial in advanced gastric/esophageal carcinoma and HNSCC has demonstrated antitumor activity [130,131]. It showed an ORR of 35.7%, including 1 CR, 12 PRs, and 2 unconfirmed PRs, in 42 patients with HNSCC [130]. Among 78 patients treated at the RP2D, the most common TRAEs were rash, hypotension, dyspnea, nausea, and dermatitis acneiform, and diarrhea [130]. It is currently being evaluated in a phase III trial of patients with PD-L1-positive HNSCC as a first-line treatment in combination with pembrolizumab compared to pembrolizumab alone (NCT06525220) and another phase III trial of patients with previously treated HNSCC compared to the investigator’s choice of chemotherapy (NCT06496178) (see Table 2).

#### 4.9.3. EGFR/CD28-Targeting BsAbs

REGN7075 is a first-in-class EGFR x CD28 BsAb that activates T cell response by bridging CD28-expressing T cells and EGFR-expressing tumor cells. The ORR was 20% and the DCR was 80% (1 CR, 2 PR, 9 SD) in combination with cemiplimab in the phase I trial of patients with advanced solid tumors [132]. REGN7075 showed clinical activity in patients with microsatellite stable metastatic colorectal cancer (mCRC) (including a patient with liver metastases); 95% of infusion-related reactions were grade 1 or 2 and were managed with premedication and split/step-up dosing. One patient experienced CRS grade 1 with fever [132]. REGN7075 is currently being evaluated in combination with cemiplimab with or without chemotherapy in a phase I/II trial of patients with advanced solid tumors (NCT04626635) as well as in a phase II trial of patients with operable stage II-IIIB NSCLC in combination with cemiplimab and chemotherapy (NCT06465329).

#### 4.9.4. EGFR/CD16a-Targeting BsAbs

AFM24 is designed as a tetravalent BsAb that binds to CD16A on NK cells and macrophages and EGFR on tumor cells to activate the innate immune system and induce ADCC and ADCP [133]. It has been investigated in a phase I/II trial in combination with atezolizumab in patients with EGFR-expressing advanced solid tumors (NCT05109442) [133]. Kim et al. reported results from the phase II trial of AFM24 in combination with atezolizumab in patients with advanced *EGFR* wild-type NSCLC who progressed on more than one prior line of therapy including platinum doublet and an ICI. One out of fifteen evaluable patients with *EGFR* wild-type NSCLC had a confirmed CR, three had PR, and seven had SD, with a DCR of 73.3%. All responders were resistant to prior ICI [134].

#### 4.9.5. EGFR/CD3-Targeting BsAbs

JANX008 and CX-904 are EGFR-targeting CD3 BiTEs that have shown activity in preclinical studies and are currently undergoing phase I clinical trials in solid tumors (NCT05783622, NCT05387265) [135].

### 4.10. VEGF-Targeting BsAbs

Ivonescimab (AK112) is a first-in-class, humanized, tetravalent BsAb targeting PD-1 and VEGF-A. Ivonescimab simultaneously blocks the binding of PD-1 to its ligand PD-L1, thereby activating immune response, and blocks the binding of VEGF-A to its receptor VEGFR2, thus interrupting tumor angiogenesis in the TME. In May 2024, ivonescimab, in combination with pemetrexed and carboplatin, received its first approval in China for the treatment of patients with *EGFR*-mutated locally advanced or metastatic non-squamous NSCLC who progressed after prior TKI therapy [13]. In the phase III randomized HARMONi-A trial in China, patients with ivonescimab plus pemetrexed and carboplatin showed better mPFS of 7.1 months compared to 4.8 months in the chemotherapy-alone group (HR: 0.46; 95% CI: 0.34–0.62; *p* < 0.001) [136]. It also showed a better ORR of 50.6% in the ivonescimab group versus 35.4% in the chemotherapy-alone group (95% CI: 5.3–26.0%; *p* = 0.006) [136]. Ivonescimab is currently under multiple clinical trials for various cancers, including ES-SCLC, BTCs, ovarian cancers, glioblastomas, HNSCC, HCC, and rectal and esophageal squamous cancers (Table 2).

PM8002 is a PD-L1 x VEGF-A which was evaluated in a phase II trial in patients with *EGFR*-mutated NSCLC who progressed on prior EGFR TKI [137]. Among 64 evaluated patients, the ORR was 54.7% with a DCR of 95.3%. The ORR was 35.7% in the PD-L1 TPS < 1% group, 56.5% in the TPS 1–49% group and 92.3% in the TPS ≥ 50% group [137]. It is currently being evaluated in combination with pemetrexed and platinum as a first-line treatment in a phase II trial of patients with unresectable malignant mesothelioma (NCT05918107), in combination with chemotherapy as a second-line treatment for neuroendocrine neoplasm (NCT05879055), and in combination with chemotherapy as a second-line treatment in SCLC (NCT06616532) in China (Table 2).

### 4.11. DLL4/VEGF-Targeting BsAbs

Delta-like ligand 4 (DLL4) and VEGF A play an essential role in angiogenesis and tumor vascularization in the TME. DLL4 is a transmembrane ligand for the Notch family of receptors, and haploinsufficiency of DLL4 results in prominent vascular defects and embryonic fatality [138]. VEGF regulates vascular permeability and angiogenesis, promoting tumor growth by increasing blood supply. DLL4 is induced by VEGF as a negative feedback mechanism and functions to prevent excessive angiogenic sprouting, thus promoting a well-differentiated vascular network formation [138]. Thus, blocking both DLL4 and VEGF simultaneously could overcome resistance to DLL4 inhibitors and provide greater antitumor activity [138].

Dilpacimab (ABT165) is a DLL4 x VEGF BsAb, with its phase I study in solid tumors showing some clinical activity (10.9% PR and 52.7% SD) and acceptable toxicity [139]. It was investigated compared to bevacizumab in combination with FOLFIRI in patients with previously treated mCRC [140]. Dilpacimab plus FOLFIRI was not well tolerated and did not provide clinical benefit to patients with mCRC compared with bevacizumab plus FOLFIRI. The ORR was 5.6% for dilpacimab plus FOLFIRI and 14.7% for bevacizumab plus FOLFIRI [140].

Navicixizumab (OMP-305B83) is another DLL4 x VEGF BsAb. Its first-in-human phase Ia trial in previously treated patients with solid tumors showed the most promising results in ovarian cancer, with four PRs in ovarian cancer patients, leading to further trials in ovarian cancer [141]. The most common TRAEs were hypertension and headache [141]. Its phase Ib trial in combination with paclitaxel in patients with platinum-resistant ovarian, primary peritoneal, and fallopian tube cancer showed an ORR of 43.2% [142]. Its subgroups showed ORRs of 33.3% in patients previously treated with bevacizumab and 64.3% in bevacizumab-naive patients, showing more promising results in bevacizumab-naive patients [142]. It is currently being evaluated in a phase II clinical trial of patients with advanced solid tumors (mCRC, gastric/GEJ cancer, triple-negative breast cancer, platinum-resistant/refractory ovarian cancer) as monotherapy or in combination with paclitaxel or irinotecan (NCT05453825). Another phase III randomized multi-center study is currently investigating its combination with paclitaxel compared to paclitaxel monotherapy in patients with platinum-resistant advanced epithelial ovarian cancer and specific biomarkers who progressed on two prior lines of treatment (NCT05043402).

CTX009 is a novel DLL4 x VEGF-A BsAb [143]. Its phase II study in combination with paclitaxel in patients with advanced BTCs showed promising efficacy, especially in the second- and third-line, settings with a 37.5% ORR, including response rates of 63.6% in the second-line setting and 15.4% in the third-line setting [143]. TRAEs of any grade were reported in 100% of patients, with grade 3 or higher TRAEs reported in 75% of patients [143]. The most common grade 3 or higher TRAEs were neutropenia, hypertension, anemia and thrombocytopenia [143]. This promising activity of CTX-009 led to its multicenter, open-label, randomized, phase II/III COMPANION-002 trial in the same patient population (NCT05506943) [144]. It is currently planned to be investigated in combination with gemcitabine, cisplatin, and durvalumab in phase I/II clinical trials of patients with unresectable or metastatic BTC (NCT06548412).

### 4.12. GD2-Targeting BsAbs

GD2, a disialoganglioside and tumor-associated antigen (TAA), is overexpressed in a variety of tumor cell types and found especially in the central nervous system and tumors of neuroectodermal origin such as neuroblastomas and melanoma [145].

Nivatrotamab is a humanized anti-GD2 x CD3 BiTE. Nivatrotamab binds to CD3 on T cells and GD2 expressed on certain tumor cells and crosslinks T cells with GD2-expressing tumor cells, thus promoting selective T-lymphocyte cytotoxicity against GD2-expressing tumor cells [146]. It was under investigation in patients with relapsed/refractory neuroblastoma, osteosarcoma and other solid-tumor cancers (NCT03860207) and in patients with relapsed and recurrent metastatic SCLC (NCT04750239). Both trials were terminated due to the manufacturer’s business priorities.

### 4.13. MUC16-Targeting BsAbs

Mucin 16 (MUC 16) is a type 1 transmembrane mucin glycoprotein that is highly expressed in epithelial ovarian and pancreatic cancer cells [147,148]. It is normally expressed in epithelia of various organs and functions as a lubricant and mucosal barrier [148].

Ubamatamab (REGN4018) is an MUC16 x CD3 BiTE that crosslinks MUC16-expressing tumor cells with CD3-expressing T cells, thus promoting T cell–mediated cytotoxic activity [147]. It has been shown to inhibit intraperitoneal ovarian tumors in preclinical studies and inhibit MUC16-expressing murine tumor cell growth. It was also found to have enhanced efficacy in combination with anti-PD1 antibodies [149]. It is currently undergoing a phase II trial in recurrent ovarian cancer or MUC16-positive endometrial cancer either alone or in combination with cemiplimab (NCT03564340) [150].

### 4.14. 5T4-Targeting BsAbs

5T4, an oncofetal antigen and an N-glycosylated protein with several leucine-rich repeats, is expressed in various cancers but rarely expressed in normal adult tissues. 5T4 plays a role in chemotaxis and epithelial–mesenchymal transition, which is associated with cancer metastasis [151].

ALG.APV-527 is a 5T4 x 4-1BB BsAb that triggers dose-dependent 4-1BB activity mediated by 5T4 crosslinking and produces antitumor responses in 5T4-expressing cells [152]. It is currently recruiting its first-in-human phase I study in patients with advanced solid tumors who failed standard treatments (NCT05934539).

### 4.15. B7-H3-Targeting BsAbs

B7-homologue 3 (B7-H3 or CD276) is a type I transmembrane protein and a member of the B7 family that is expressed on both tumor and immune cells. It has both costimulatory and coinhibitory roles in the immune system and promotes tumorigenesis, including tumor cell growth, invasion and metastasis [4,153].

XmAb808 is a tumor-selective XmAb 2 + 1 BsAb targeting B7-H3 on tumor cells and CD28 co-receptor on T cells, thus designed to activate CD28 only in the presence of tumor cells. Bivalent and high-avidity B7H3 binding directs XmAb808 to cancer cells with high levels of B7H3 expression, while low-affinity binding to CD28 prevents superagonism of T cells [154]. Currently, its phase I trial in combination with pembrolizumab in advanced solid tumors is ongoing to evaluate its safety and efficacy (NCT05585034) [154].

CC-3 is an IgG-based B7-H3 and CD3 BiTE. Given that B7-H3 is expressed in different sarcoma subtypes and CRC, it would be a potential therapeutic option [155]. It has been shown to promote T cell activation with reduced undesired cytokine release in an in vitro study and potent antitumor activity in an in vivo study with immunocompromised mice [156]. Currently its phase I study is recruiting patients with CRC (NCT05999396).

### 4.16. TGF-β/PDL1-Targeting BsAbs

TGF-β (transforming growth factor-beta) is a multifunctional cytokine which has essential roles in cell growth and apoptosis, and it acts as tumor suppressor. However, it can become oncogenic in tumor cells as it loses its antiproliferative activity [157].

Bintrafusp alfa is a first-in-class TGF-β fused to the PD-L1-targeting IgG1 monoclonal antibody. Its phase I trial in 83 patients with advanced NSCLC with resistance or refractory to prior PDL1 treatment showed four PRs and nine SDs [158]. The phase I trial of bintrafusp in advanced HNSCC showed an ORR of 13%, with four PRs and four SDs, with a DCR of 34% [159]. Its phase II trial evaluated 146 patients with recurrent or metastatic cervical cancer who progressed on prior platinum-based chemotherapy, and bintrafusp showed an ORR of 21.9%, and 59.4% of responders had a durable response of 6 months or more. The most common TRAEs were anemia, rash, hypothyroidism and pruritus [160]. A randomized phase III trial of bintrafusp alfa as first-line treatment in PD-L1-high advanced NSCLC showed PFS of 7.0 months compared to 11.1 months in the pembrolizumab group (HR = 1.201, 95% CI: 0.796–1.811) [161]. Bintrafusp alfa is currently under several clinical trials including a phase II trial in patients with advanced sarcoma in combination with doxorubicin (NCT04874311), a phase II trial in patients with thymoma and thymic carcinoma (NCT04417660), and in combination with chemoradiation in patients with esophageal or GEJ squamous cell carcinoma (NCT04481256).

SHR-1701 is a BsAb composed of a monoclonal antibody against PD-L1 fused to the extracellular domain of TGF-β [162]. Its phase Ib study of patients with metastatic nasopharyngeal carcinoma showed an ORR of 33.3% and DCR of 53.3% in arm 1 (prior platinum-based chemotherapy) and an ORR of 4.2% and DCR of 25.0% in arm 2 (prior platinum-based chemotherapy and PD-1/PD-L1 antibody treatment). The most common grade 3 TRAEs were anemia and hemoptysis and occurred in 18.5% of patients [162]. Its combination with famitinib (multi-kinase TKI) in patients with previously treated advanced BTC or pancreatic ductal adenocarcinoma showed an ORR of 28% (two CRs), DCR of 80%, mPFS of 5.1 months and mOS of 16 months in the BTC cohort and an ORR of 15% (two CRs), DCR of 60%, mPFS of 2.1 months and mOS of 5.3 months [163]. It was also evaluated in a phase I study of metastatic cervical cancer and as neoadjuvant treatment with or without chemotherapy in stage III NSCLC [164,165]. It is currently being investigated in various clinical trials in China, including a phase II trial in combination with temozolomide in the treatment of advanced melanoma (NCT05106023) and a phase II trial in combination with chemoradiation in locally advanced rectal cancer (NCT05300269).

### 4.17. Other BsAbs

There are numerous other BsAbs currently under investigation, including ENPP3-targeting, MSLN-targeting, PRAME-targeting and CDH17-targeting BsAbs, among others. ENPP3, ectonuclotide pyrophosphatase/phosphodiesterase family number 3 or CD203c, is a transmembrane glycoprotein that plays an essential role in cellular processes and cell signaling. It is selectively expressed in tumor cells including RCC, lung adenocarcinoma, endometrioid uterine and ovarian cancers, CRC, and HCC [166]. JNJ-87890387 and XmAb-819 are ENPP3xCD3 BiTEs that have shown activity and safety in preclinical studies and are currently in phase I trials in advanced solid tumors (NCT06178614, NCT05433142) [166,167].

MSLN, mesothelin, is a protein involved in cell adhesion function in normal cells but overexpressed in various cancer cells, including mesothelioma and lung, pancreas and CRC [168]. JNJ-79032421 is an MSLN x CD3 which is currently being evaluated in a phase I clinical trial of advanced solid tumors (NCT06255665).

PRAME, preferentially expressed antigen in melanoma, is an intracellular protein and a member of the CUL2 ubiquitin ligase complex involved in protein degradation, and it is normally expressed in the testis [169]. It becomes overexpressed in multiple solid tumors and hematological malignancies [170]. IMA402 targets HLA-A*02-presented peptide derived from PRAME x CD3 on T cells and is currently under a phase I/II trial of patients with recurrent or refractory solid tumors (NCT05958121) [170]. Another PRAME x CD3-targeting BsAb, IMC-F106C, is currently under a phase III trial of patients with treatment-naïve HLA-A*02:01-positive advanced melanoma in combination with nivolumab (PRISM-MEL-301, NCT06112314).

## 5. Challenges in Clinical Utilization of BsAbs in Solid Tumors

BsAbs have demonstrated remarkable promise in the treatment of solid tumors by facilitating immune effector cell recruitment and enhancing tumor cytotoxicity. However, some challenges have limited their clinical translation, principally on-target, off-tumor toxicity and CRS, which have resulted in unintended tissue damage [171]. Furthermore, tumor cells employ multiple immune evasion strategies to suppress T-cell activation and escape immune surveillance, including loss of antigen expression, antigen internalization, and antigen shedding. The immunosuppressive nature of the TME, along with concurrent infections, can further exacerbate the adverse effects associated with BsAb therapy [172].

### 5.1. Toxicities

#### 5.1.1. On-Target, Off-Tumor Toxicities

On-target, off-tumor toxicity is a phenomenon wherein a BsAb, designed to target a TAA, also binds to the same antigen on normal tissues, leading to unintended T cell activation and tissue damage [171,173]. This can result in various toxicities, including immune-related adverse events. Although on-target, off-tumor effects can also occur in the context of hematological malignancies, the clinical risk of severe adverse events is generally lower compared to solid tumors [174].

Targets of BsAbs developed for hematological malignancies, including CD19, CD20, CD33, CD38 (also known as ADPRC1), CD123, and B-cell maturation antigen (BCMA, also known as TNFRSF17), are commonly expressed on normal B and plasma cells [171]. However, depletion of these B-lineage cells is usually well tolerated, provided that hematopoietic stem cells remain unaffected, allowing for regeneration of the hematopoietic system and thereby minimizing the clinical consequences of B-cell loss [174].

In contrast, many BsAbs for solid-tumor target antigens, such as HER2, EGFR variant III (vIII), PSMA and EpCAM, are also expressed at low levels in essential normal tissues [173]. This antigen distribution raises the risk of serious adverse events. For instance, in studies on cynomolgus monkeys treated with MGD007, evidence of gastrointestinal (GI) toxicity was observed [175]. This toxicity is believed to be due to the drug’s on-target engagement with the gpA33 antigen on intestinal cells. Similarly, EGFR-targeting BsAbs like amivantamab have been associated with dermatologic toxicities, including rash and paronychia, reflective of EGFR’s physiological role in maintaining skin homeostasis [22].

Achieving high selectivity and specificity remains a major challenge in the development of BsAbs for solid tumors [172]. Several strategies have been developed to enhance the selectivity of BsAbs for malignant cells:

- Antigen density discrimination: BsAbs can be engineered to preferentially bind to cells with high antigen density, sparing normal tissues with low antigen expression [176].
- Dual-antigen recognition: By requiring simultaneous binding to two distinct TAAs (e.g., HER2 and HER3, or HER3 and EGFR), BsAbs can increase tumor specificity and minimize the risk of off-tumor activation [172,177,178].
- Affinity modulation: Tuning the binding affinity of BsAbs toward TAAs is critical; lower-affinity antibodies can preferentially bind tumor cells with abundant antigens while avoiding low-expression normal tissues. However, excessive affinity reduction can compromise therapeutic efficacy, highlighting the need for precise molecular engineering [179].
- Switchable BsAb formats: Recent advances have introduced BsAbs that are activated only in the presence of tumor-specific enzymatic activities, such as tumor-associated proteases cleaving an inhibitory domain. These “prodrug” designs further improve tumor selectivity by restricting T-cell activation to the TME [180].

In T-cell-redirecting BsAbs specifically, careful modulation of antigen-binding strength is essential. If the binding affinity is too high, there is a risk of targeting healthy cells expressing low levels of TAAs. Conversely, excessively low affinity may reduce the therapeutic potency of T-cell engagement and cytotoxicity. Ongoing innovations aim to balance these factors to maximize antitumor activity while minimizing off-tumor toxicity [172].

#### 5.1.2. Cytokine Release Syndrome

CRS does not occur in all BsAbs but it represents a major dose-limiting toxicity associated with T-cell-engaging BsAbs (BiTE). It results from widespread immune activation and the massive release of pro-inflammatory cytokines, including interleukin-6 (IL-6), interferon-gamma (IFN-γ), and tumor necrosis factor-alpha (TNF-α) [181]. The clinical spectrum of CRS can vary widely, ranging from mild symptoms such as fever, fatigue, and myalgias to severe, life-threatening manifestations including hypotension, hypoxia, and multiorgan dysfunction [182]. Severe CRS events pose significant challenges for clinical management and are a critical consideration in BiTE therapy design.

Mechanistically, CRS is initiated when BiTEs simultaneously engage tumor cells and CD3-expressing T cells, leading to immunological synapse formation, activation of the T-cell receptor (TCR) complex, and downstream cytokine cascades [181]. Unlike targeted killing that is localized within the TME, BsAb-induced T-cell activation can trigger systemic immune responses, particularly in settings of high tumor burden or widespread antigen expression, or when the BsAb format possesses a prolonged half-life [183].

The pioneering BsAb catumaxomab demonstrated promising efficacy in the treatment of malignant ascites. However, its strong immunogenicity led to significant hepatotoxicity and provoked high-grade CRS when off-target immune activation occurred. These adverse events ultimately contributed to the withdrawal of catumaxomab from the market in 2017 [1].

Multiple strategies have been developed to mitigate CRS risk while maintaining therapeutic efficacy:

- Step-up dosing regimens involve administering a lower priming dose followed by gradual escalation to therapeutic levels, allowing for progressive T-cell desensitization and reducing the peak cytokine surge. Although step-up dosing is not mandated by prescribing labels, clinicians should consider monitoring patients for 1 to 2 h following administration when dosing occurs in the outpatient setting. If signs or symptoms of CRS develop, prompt admission to the hospital or transfer to the emergency department should be considered based on clinical severity [184].
- Pre-medications, including corticosteroids and IL-6 receptor antagonists such as tocilizumab, are employed prophylactically or reactively to dampen the inflammatory response without abrogating antitumor activity [185]. Immunosuppressive therapies should be considered to attenuate the immune response in cases of severe CRS. Accurate grading of these toxicities is critical, as it guides clinicians in initiating appropriate interventions. Mild-to-moderate CRS can often be managed with supportive care measures, including intravenous fluids, antihistamines, and antipyretics. In contrast, patients who develop severe CRS require close monitoring and management in an intensive care unit setting [184]. Treatment with dexamethasone at a dose of 8 mg intravenously or orally every 8 h for 3 days is recommended, followed by a gradual steroid taper over the subsequent 4 days. If patients exhibit an inadequate clinical response to corticosteroid therapy or have grade 3 to 4 CRS, administration of tocilizumab, an IL-6 receptor antagonist, at a dose of 8 mg/kg intravenously is indicated. Tocilizumab has demonstrated efficacy in reversing severe manifestations of CRS by targeting the IL-6-mediated inflammatory cascade [184,186].

Despite these advances, balancing the reduction of CRS risk without compromising BsAb-mediated antitumor cytotoxicity remains an ongoing clinical challenge. CRS severity and incidence continue to serve as critical determinants in the clinical success and regulatory approval of BsAb therapies, especially in the treatment of solid tumors.

### 5.2. Tissue Penetration and Resistance to BsAb Therapy

#### 5.2.1. Tumor Microenvironment

Solid tumors pose formidable physical barriers to effective BsAb distribution and action. The TME, characterized by elevated interstitial fluid pressure, dense extracellular matrix deposition, and abnormal vasculature, restricts the infiltration and diffusion of therapeutic agents [187].

BsAbs, although smaller than full-length IgG antibodies, still face significant challenges related to their molecular size and binding-site accessibility. Early binding of BsAbs to peripheral tumor cells, known as the binding-site barrier effect, can prevent deeper penetration into the tumor core, while normal tissues with low-level antigen expression can act as antigen sinks, reducing antibody availability [188,189].

Strategies to overcome these barriers include engineering smaller BsAb constructs, such as single-chain variable fragments (scFvs), transiently degrading the tumor stroma using agents like hyaluronidase, and modulating pharmacokinetics to enhance tissue retention while minimizing systemic clearance. However, optimizing intratumoral delivery remains a significant challenge [172].

#### 5.2.2. Loss of Target Antigen Expression and Resistance to BsAb Therapy

Loss or downregulation of target antigen expression is a key mechanism of immune escape in cancers treated with T-cell-redirecting BsAbs [172]. BiTEs require the presence of specific TAAs on the surface of malignant cells to mediate recruitment, activation of T cells and subsequent tumor lysis. Tumors under immunological pressure may downregulate or entirely lose the targeted TAA, thereby evading T-cell-mediated cytotoxicity and resulting in subsequent therapeutic failure [187]. For instance, Barakzai et al. evaluated cells derived from ascitic fluid of patients after progression on BiTEs and found that tumor cells that relapsed after BiTE therapy frequently showed loss of target antigens, which correlated with reduced ex vivo cytotoxicity despite preserved T cell function [190].

**Figure 2:**
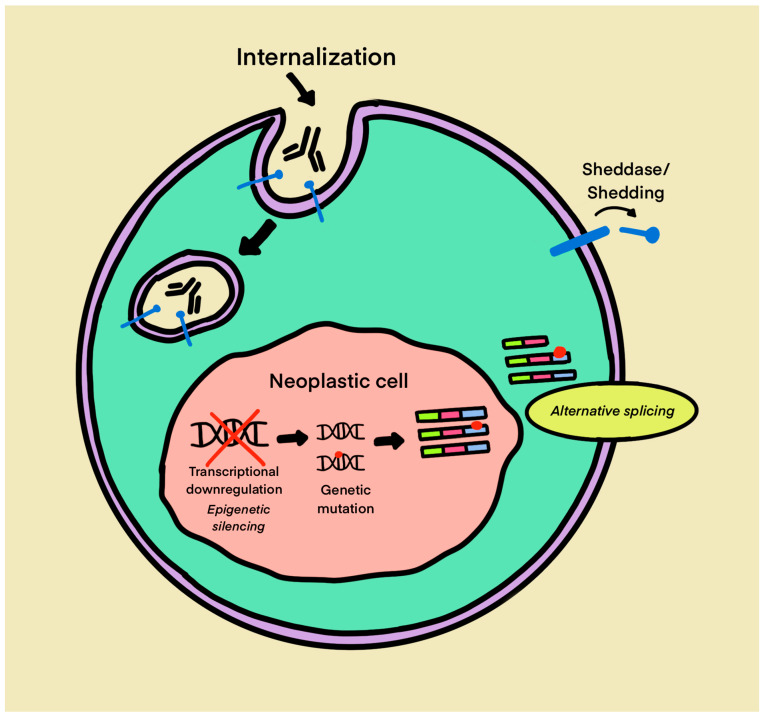
Resistance to BsAbs due to loss of antigen expression via internalization (endocytosis), proteolytic shedding of the target, genetic mutations in genes encoding the target antigen, epigenetic regulation (silencing of gene expression via DNA methylation or histone modification leading to transcriptional downregulation), alternative splicing (splice variants that do not present epitopes recognized by BsAbs), or clonal selection (tumor cells lacking the target antigen of BsAb therapy can survive and expand, leading to a resistant population).

Tumor antigen loss can occur through several mechanisms, including antigen shedding, internalization (endocytosis), mutations leading to epitope alteration, and antigenic drift-like evolution under immune pressure [191] (see Figure 2). Particularly in solid tumors such as gastrointestinal cancers, melanoma, and gliomas, the scarcity of truly tumor-specific antigens and the intrinsic heterogeneity of tumor cells complicate target selection [192].

As tumors adapt, therapies focused on a single antigen may prove insufficient to sustain durable responses. Future directions will likely involve combination BsAb therapies or multi-specific designs that target multiple antigens simultaneously, aiming to minimize resistance development and maximize therapeutic efficacy. For instance, tetraspecific BsAbs that target EGFR, HER2, HER3 and VEGF could demonstrate more effective antitumor activity and disrupt drug resistance induced by their parental BsAbs [193].

#### 5.2.3. Anti-Drug Antibodies

Given their large and complicated structures, BsAbs can induce a persistent humoral response, resulting in neutralizing anti-drug antibodies against the variable regions of BsABs and interrupting target antigen engagement [1]. It can cause alteration of drug pharmacokinetics or complement-mediated or infusion-related reactions. The incidence of formation of anti-drug antibodies is higher with BsAbs targeting T cells or antigen-presenting cells compared to those targeting B cells [1]. As we discussed previously, this has been the reason for the drug termination of PSMA-targeting BsAbs (pasotuxizumab, acapatamab) and PD-L1 x TIM-3 targeting LY3415244.

## 6. Conclusions

The treatment landscape of cancer has developed exponentially over the last decade with several drug approvals and personalized therapy. Among those innovative cancer treatments, BsAbs have been leading the way, especially in hematological malignancies. Despite their late start in the game, BsAbs in solid tumors have recently shown potential, with five BsAbs approved in the United States in the past four years.

There are several other BsAbs currently in clinical trials, but there are still challenges due to the heterogeneity of solid malignancies, tissue selectivity and specificity, drug penetrance due to stromal barriers, resistance due to tumor antigen loss, on-target, off-tumor toxicities and CRS associated with BiTEs. BsAbs with specific targets like EGFR, HER2, and NRG1 have shown promising potential and led the way for personalized precision medicine. Future directions of BsAbs involve combination BsAb therapies or multi-specific designs that target multiple antigens simultaneously, aiming to minimize resistance development and maximize therapeutic efficacy, optimizing intratumoral delivery and selectivity for malignant cells to minimize off-target toxicities, and using biomarkers to predict response and tailor personalized cancer treatment. Current trials are investigating the use of BsAbs in combination with chemotherapy, TKIs and other immunotherapy to optimize their clinical utilization. Therefore, the future of BsAbs in solid tumors seems very promising, and we have yet to see their full potential.

## Figures and Tables

**Table 1:** Current FDA-approved BsAbs in the United States and trials leading to their approvals.

Trial	Patient Population	Number of Patients	Treatment Arm	mOS (Months)	mPFS (Months)	ORR (%)	mDOR (Months)	FDA Approval
IMCgp100-202 Phase III [18]	Previously untreated metastatic uveal melanoma with HLA-A*02:01	252	tebentafusp	21.6	3.4	11	11.1	25 January 2022
		126	investigator choice (control group): pembrolizumab, ipilimumab or dacarbazine	16.9	2.9	5	9.7	
PAPILLON Phase III [21]	Advanced NSCLC with EGFR exon 20 insertions without prior systemic treatment	153	amivantamab-chemotherapy	NE	11.4	73	9.7	1 March 2024
		155	chemotherapy	24.4	6.7	47	4.4	
CHRYSALIS Phase I [22]	Advanced or metastatic NSCLC with EGFR exon 20 insertions who progressed on or after platinum-based chemotherapy.	81	amivantamab	22.8	8.3	40	11.1	21 May 2021
MARIPOSA Phase III [23,24]	Locally advanced or metastatic NSCLC with EGFR exon 19 deletions or exon 20 L858R mutations	429	amivantamab-lazertinib	NE	23.7	86	25.8	20 August 2024
		429	osimertinib	36.7	16.6	85	16.8	
		216	lazertinib	-	-	-	-	
DeLLphi-301 Phase II [25]	Extensive-stage SCLC with disease progression after platinum-based chemotherapy	100 (10 mg)	tarlatamab	14.3	4.9	40	-	16 May 2024
HERIZON-BTC-01 Phase IIb [26,27]	HER2-amplified, unresectable, locally advanced or mBTC patients who progressed on prior gemcitabine therapy	80	zanidatamab	15.5	5.5	41.3	12.9	20 November 2024
eNRGy Phase II [28]	Advanced or metastatic *NRG1* fusion-positive NSCLC patients who had disease progression following standard-of-care treatment	65	zenocutuzumab	-	-	34	12.9	4 December 2024
eNRGy Phase II [29]	Advanced or metastatic *NRG1* fusion-positive pancreatic adenocarcinoma patients who had disease progression following standard-of-care treatment	27	zenocutuzumab	-	-	44	9.1	4 December 2024

NE—not estimable.

**Table 2:** Selected current ongoing clinical trials of BsAbs.

Target	Agent	Phase	Clinical Trial	Status	Conditions
Gp100 x CD3	Tebentafusp	II	NCT06070012	R	HLA-A*02:01-positive previously untreated metastatic uveal melanoma
		II	NCT06246149	R	HLA-A*02:01-positive high-risk uveal melanoma following definitive treatment (as adjuvant treatment)
		II	NCT06414590	R	Large surgically unresectable (other than complete enucleation of eye) primary uveal melanoma (as neoadjuvant treatment)
		III	NCT05549297	R	HLA-A*02:01-positive patients with previously treated advanced melanoma (TEBE-AM)
EGFR x cMET	Amivantamab	I/II	NCT06385080	R	Recurrent/metastatic head and neck squamous cell carcinoma (HNSCC)
		I/II	NCT05845671	R	Advanced NSCLC harboring *ALK*, *ROS1* and *RET* gene mutations (in combination with TKI)
		II	NCT05299125	A, NR	Recurrent/metastatic NSCLC with *EGFR* mutations (in combination with lazertinib and pemetrexed)
		III	NCT06662786	R	*KRAS/NRAS/BRAF* wild-type unresectable or metastatic left-sided CRC (in combination with mFOLFOX6 or FOLFIRI) as first line
		III	NCT06750094	R	Previously treated *KRAS/NRAS/BRAF* wild-type colon cancer (in combination with FOLFIRI)
		I/II	NCT06083857	R	*MET*-altered NSCLC (in combination with tepotinib
		I	NCT06632236	R	High-grade malignant brain tumors with EGFR amplification
		I/II	NCT05379595	R	Advanced or metastatic CRC
		II	NCT06667076	R	*EGFR*-mutated locally advanced or metastatic NSCLC (in combination with lazertinib compared to amivantamab plus platinum-based chemotherapy)
DLL3 x CD3	Tarlatamab	III	NCT06211036	R	ES-SCLC following treatment with platinum, etoposide and durvalumab
		III	NCT06117774	R	LS-SCLC who have not progressed following concurrent chemoradiation
		II	NCT06788938	R	Advanced DLL3-expressing tumors including neuroendocrine neoplasms
HER2 x HER3	Zenocutuzumab	II	NCT02912949	A, NR	Solid tumors harboring an *NRG1* fusion
HER2 (2 distinct domains)	Zanidatamab	III	NCT06282575	R	Advanced HER2-positive BTC (in combination with chemotherapy with or without PD-1/PDL1 inhibitor)
		II	NCT06043427	R	HER2-positive advanced GEJ adenocarcinoma who failed at least one prior trastuzumab-containing regimen (in combination with paclitaxel and ramucirumab)
		II	NCT05035836	R	Early-stage HER2-positive breast cancer
		III	NCT06435429	R	Metastatic HER2-positive breast cancer
HER2 x CD3	Runimotamab (RG6194)	I	NCT03448042	A, NR	Metastatic HER2-positive breast cancer
HER2 (2 distinct domains)	KN026	II	NCT05985707	NYR	HER2-positive colorectal and biliary carcinoma
		III	NCT06747338	NYR	Early or locally advanced HER2-positive breast cancer (in combination with HB1801 as neoadjuvant treatment)
		II/III	NCT05427383	R	HER2-positive gastric cancer patients who failed first-line therapy
HER2 x 4-1BB	YH32367 (ABL105)	I/II	NCT05523947	R	HER2-positive locally advanced or metastatic solid tumors
HER3 x EGFR	Izalontamab (SI-B001)	III	NCT05943795	R	Previously treated NSCLC (adenocarcinoma and squamous cell carcinoma) in combination with docetaxel
		II	NCT06668961	R	Recurrent or metastatic HNSCC (in combination with SI-B003 and platinum-based chemotherapy)
		II	NCT05054439	R	Recurrent and metastatic HNSCC (in combination with paclitaxel)
PSMA x CD3	Acapatamab (AMG 160)				Discontinued
PSMA x CD3	AMG 340	I	NCT04740034	Completed	mCRPC
PSMA x CD3	JNJ-081/JNJ63898081	I	NCT03926013	Completed	Advanced solid tumors
PSMA x CD28	REGN5678	I/II	NCT03972657	R	mCRPC and other tumors (in combination with cemiplimab)
CLDN18.2 x CD3	Gresonitamab (AMG 910)	I	NCT04260191	Terminated	Claudin 18.2-positive gastric and GEJ adenocarcinoma
CLDN18.2 x CD3	AZD5863	I/II	NCT06005493	R	Advanced or metastatic solid tumors
CLDN18.2 x CD3	LNF2007	I	NCT06752447	NYR	Advanced solid tumors
CLDN18.2 x CD3	ASP2138	I	NCT05365581	R	CLDN 18.2-positive advanced gastric/GEJ or pancreatic cancer
CLDN18.2 x PD-L1	Q-1802	I	NCT04856150	R	CLDN18.2-expressing advanced or metastatic solid tumors
		I/II	NCT05964543	R	Advanced or recurrent metastatic CLDN18.2-positive primary gastric/GEJ adenocarcinoma (in combination with XELOX)
CEA x CD3	Cibisatamab (RO6958688)	I/II	NCT03337698	A, NR	Metastatic NSCLC
CEA x CD3	MEDI565 (AMG211)	I	NCT02291614	Terminated	Advanced gastrointestinal cancer
EpCAM x CD3	Catumaxomab				Discontinued
EpCAM x CD3	Solitomab (AMG 110)				No current clinical trials
EpCAM x CD3	BA3182	I	NCT05808634	R	Advanced adenocarcinoma
EpCAM x 4-1BB	BNT314/GEN1059	I/II	NCT06150183	R	Metastatic or advanced malignant solid tumors
GPC3 x CD3	ERY974	I	NCT05022927	A, NR	Locally advanced or metastatic HCC
GPC3 x CD3	SAR-4442000	I/II	NCT05450562	A, NR	Advanced solid tumors (alone or in combination with atezolizumab)
HLA-G x CD3	JNJ-78306358				No current clinical trials
PD-1 x CTLA4	Cadonilimab (AK104)	II	NCT05932212	R	Recurrent or metastatic vulvar cancer (alone or in combination with chemotherapy)
		III	NCT06566755	R	RAS-mutated or right-sided metastatic microsatellite stable CRC (in combination with chemotherapy and bevacizumab)
		II	NCT06448910	R	Locally advanced unresectable stage III NSCLC (concurrent chemoradiotherapy)
		III	NCT05489289	R	Adjuvant therapy in HCC with high-risk recurrence after curative resection
		II/III	NCT06241599	R	Recurrent or metastatic nasopharyngeal carcinoma (in combination with chemotherapy)
PD-1 x CTLA4	Volrustomig (MEDI5752)	III	NCT06097728	R	Unresectable pleural mesothelioma (in combination with carboplatin and pemetrexed)
		III	NCT06079671	R	High-risk locally advanced cervical cancer (FIGO stage IIIC to IVA)
		I	NCT05821231	R	Metastatic soft tissue sarcoma (in combination with radiation)
		III	NCT06129864	R	Unresected locally advanced HNSCC patients who have not progressed after receiving definitive concurrent chemoradiotherapy
		III	NCT05984277	R	mNSCLC and PD-L1 < 50% (in combination with chemotherapy) (eVOLVE-Lung02 trial)
PD-1 x CTLA4	Lorigerlimab (MGD019)	II	NCT05848011	A, NR	mCRPC (in combination with docetaxel)
		II	NCT05475171	R	Advanced or metastatic cervical cancer
		II	NCT06730347	R	Previously treated patients with platinum-resistant ovarian cancer or clear-cell gynecologic cancer
PD-L1 x CTLA4	Erfonrilimab (KN046)	II/III	NCT06020352	R	Neoadjuvant therapy in stage IB-IIIB NSCLC (in combination with axitinib)
		II	NCT06099821	R	MSI-H gastrointestinal cancers resistant to PD-1/PDL1 (in combination with regorafenib or apatinib)
PD-L1 x CTLA4	Vudalimab (XmAb20717)	II	NCT05005728	R	mCRPC who progressed on prior therapy (alone or in combination with chemotherapy)
		I/II	NCT06173505	R	Advanced NSCLC
PD-1 x TIGIT	Rilvegostomig (AZD2936)	III	NCT06627647	R	Metastatic NSCLC patients whose tumors express PD-L1 (≥1%)
PD-1 x IL-2	IBI363	I	NCT05460767	R	Advanced solid tumors or lymphoma
		II	NCT06281678	R	Advanced solid malignancies (melanoma, NSCLC, CRC, RCC)
		II	NCT06081920	R	Advanced melanoma
		I	NCT06610799	R	Advanced or metastatic gastric/GEJ cancer (in combination with capecitabine and oxaliplatin)
		II	NCT06797297	R	Unresectable or metastatic mucosal or acral melanoma without prior systemic therapy
PD-1 x ICOS	XmAb23104			Terminated	No current clinical trials
PD-1× LAG-3	Tebotelimab (MGD013)			Terminated	No current clinical trials
PD-L1 x LAG-3	ABL501				No current clinical trials
PD-L1 x PD-1	LY3434172 (IBI318)	II	NCT04777084	R	Advanced NSCLC patients who failed first-line PD-1/PD-L1 inhibitor therapy, advanced NSCLC with *EGFR*-sensitive mutation/*ALK* fusion after EGFR-TKI/ALK-TKI treatment resistance, and advanced NSCLC with negative PD-L1 expression *EGFR, ALK and ROS1* wild type
PD-L1 x PD-1	CTX8371	I	NCT06150664	R	Advanced malignancies
PD-L1/TIM-3	LY3415244	I	NCT03752177	Terminated	Advanced solid tumors
CD47 x PD-L1	IBI322	II	NCT05296603	R	ES-SCLC patients who failed first-line PD-L1 inhibitors (in combination with lenvatinib)
PD-L1 x 4-1BB	FS222	I	NCT04740424	R	Previously treated patients with advanced tumors
PD-L1 x 4-1BB	MCLA145				No current clinical trials
PD-L1 x 4-1BB	ATG101	I	NCT04986865	R	Metastatic/advanced solid tumors and mature B cell non-Hodgkin lymphoma
PD-L1 x 4-1BB	ABL503	I	NCT04762641	R	Locally advanced or metastatic solid tumors
PD-L1 x OX40	KN051	I	NCT05309512	Terminated	Advanced solid tumors
PD-L1 x OX40	EMB-09	I	NCT05263180	R	Advanced or metastatic solid tumors
EGFR x cMET	Bafisontamab (EMB01)	I/II	NCT05498389	NYR	*EGFR* mutant NSCLC who progressed on standard treatment
		I/II	NCT05176665	R	Advanced or metastatic gastrointestinal cancer (gastric cancer, HCC, cholangiocarcinoma and CRC)
		I/II	NCT03797391	R	Previously treated *EGFR* and/or *cMET* mutated advanced or metastatic solid tumors
EGFR x cMET	MCLA-129	I/II	NCT04868877	R	Advanced NSCLC and other solid tumors
EGFR x LGR5	Petosemtamab (MCLA158)	III	NCT06525220	R	PD-L1-positive HNSCC
		III	NCT06496178	R	Previously treated HNSCC
EGFR x CD28	REGN7075	II	NCT06465329	R	Operable stage II-IIIB NSCLC
EGFR x CD16A	AFM24	I/II	NCT05109442	R	EGFR-expressing advanced solid tumors (in combination with atezolizumab)
EGFR x CD3	JANX008	I	NCT05783622	R	Advanced or metastatic solid tumor malignancies
EGFR x CD3	CX-904	I	NCT05387265	R	Advanced solid tumors
VEGFA x PD-1	Ivonescimab (AK112)	III	NCT06767514	R	Metastatic NSCLC with high PD-L1
		II	NCT06567314	R	Cutaneous squamous cell carcinoma
		II	NCT06925724	R	Endometrial and cervical cancers
		II	NCT06848842	R	Unresectable CRC
		II	NCT06375486	R	Unresectable HCC
VEGFA x PD-1	PM8002	II	NCT05918107	R	Unresectable malignant mesothelioma
		II	NCT05879055	R	Previously treated neuroendocrine neoplasm
		III	NCT06616532	R	Previously treated SCLC (in combination with paclitaxel)
DLL4 x VEGF	Dilpacimab (ABT165)				No current clinical trials
DLL4 x VEGF	Navicixizumab (OMP-305B83)	II	NCT05453825	Unknown	Advanced solid tumors
		III	NCT05043402	Unknown	Platinum-resistant advanced epithelial ovarian cancer and specific biomarkers who progressed on 2 prior lines
DLL4 x VEGF	CTX009 (ABL001)	II/III	NCT05506943	A, NR	Unresectable advanced, metastatic or recurrent BTCs (in combination with paclitaxel)
		I/II	NCT06548412	R	Unresectable or metastatic BTC (in combination with gemcitabine, cisplatin and durvalumab)
GD2 x CD3	Nivatrotamab	I/II	NCT03860207	Terminated	Relapsed/refractory neuroblastoma, osteosarcoma and other solid tumor cancers
		I/II	NCT04750239	Terminated	Relapsed and recurrent metastatic SCLC
MUC16 x CD3	Ubamatamab (REGN4018)	I/II	NCT03564340	R	Recurrent ovarian cancer or MUC16-positive cancers
5T4 x 4-1BB	ALG.APV-527	I/II	NCT05934539	R	Advanced solid tumors who failed standard treatments
B7-H3 x CD28	XmAb808	I	NCT05585034	A, NR	Advanced solid tumors (in combination with pembrolizumab)
B7-H3 x CD3	CC-3	I	NCT05999396	R	Metastatic CRC
TGF-β x PDL1	Bintrafusp alfa	II	NCT04874311	R	Advanced sarcoma
		II	NCT04417660	R	Thymoma and thymic carcinoma
		NA	NCT04481256	R	Esophageal or GEJ squamous cell carcinoma
TGF-β x PDL1	SHR-1701	II	NCT05106023	R	Advanced melanoma (in combination with temozolomide)
		II	NCT05300269	R	Locally advanced rectal cancer
ENPP3 x CD3	XmAb-819	I	NCT05433142	R	Advanced solid tumors
ENPP3 x CD3	JNJ-87890387	I	NCT06178614	R	Advanced solid tumors
MSLN x CD3	JNJ-79032421	I	NCT06255665	R	Advanced solid tumors
PRAME x CD3	IMA-402	I/II	NCT05958121	R	Recurrent or refractory solid tumors
PRAME x CD3	IMC-F106C	III	NCT06112314	R	Treatment naive HLA-A*02:01-positive advanced melanoma

Retrieved from https://clinicaltrials.gov/; accessed on 20 April 2025. R—recruiting; A, NR—active, not recruiting. CRC—colorectal cancer, HCC—hepatocellular carcinoma, RCC—renal cell cancer, mCRPC—metastatic castration resistant prostate cancer, mFOLFOX—modified fluorouracil and oxaliplatin, FOLFIRI—fluorouracil and irinotecan, XELOX—capecitabine and oxaliplatin.
